# Functional analysis reveals G/U pairs critical for replication and trafficking of an infectious non-coding viroid RNA

**DOI:** 10.1093/nar/gkaa100

**Published:** 2020-02-21

**Authors:** Jian Wu, Cuiji Zhou, James Li, Chun Li, Xiaorong Tao, Neocles B Leontis, Craig L Zirbel, David M Bisaro, Biao Ding

**Affiliations:** Department of Molecular Genetics, Center for Applied Plant Sciences, Center for RNA Biology, and Infectious Diseases Institute, The Ohio State University, Columbus, OH 43210, USA; Graduate Program in Molecular, Cellular, and Developmental Biology, The Ohio State University, Columbus, OH 43210, USA; Department of Molecular Genetics, Center for Applied Plant Sciences, Center for RNA Biology, and Infectious Diseases Institute, The Ohio State University, Columbus, OH 43210, USA; Department of Molecular Genetics, Center for Applied Plant Sciences, Center for RNA Biology, and Infectious Diseases Institute, The Ohio State University, Columbus, OH 43210, USA; Department of Plant Pathology, Nanjing Agricultural University, Nanjing 210095, China; Department of Plant Pathology, Nanjing Agricultural University, Nanjing 210095, China; Department of Chemistry, Bowling Green State University, Bowling Green, OH 43403, USA; Department of Mathematics and Statistics, Bowling Green State University, Bowling Green, OH 43403, USA; Department of Molecular Genetics, Center for Applied Plant Sciences, Center for RNA Biology, and Infectious Diseases Institute, The Ohio State University, Columbus, OH 43210, USA; Graduate Program in Molecular, Cellular, and Developmental Biology, The Ohio State University, Columbus, OH 43210, USA; Department of Molecular Genetics, Center for Applied Plant Sciences, Center for RNA Biology, and Infectious Diseases Institute, The Ohio State University, Columbus, OH 43210, USA; Graduate Program in Molecular, Cellular, and Developmental Biology, The Ohio State University, Columbus, OH 43210, USA

## Abstract

While G/U pairs are present in many RNAs, the lack of molecular studies to characterize the roles of multiple G/U pairs within a single RNA limits our understanding of their biological significance. From known RNA 3D structures, we observed that the probability a G/U will form a Watson–Crick (WC) base pair depends on sequence context. We analyzed 17 G/U pairs in the 359-nucleotide genome of *Potato spindle tuber viroid* (PSTVd), a circular non-coding RNA that replicates and spreads systemically in host plants. Most putative G/U base pairs were experimentally supported by selective 2′-hydroxyl acylation analyzed by primer extension (SHAPE). Deep sequencing PSTVd genomes from plants inoculated with a cloned master sequence revealed naturally occurring variants, and showed that G/U pairs are maintained to the same extent as canonical WC base pairs. Comprehensive mutational analysis demonstrated that nearly all G/U pairs are critical for replication and/or systemic spread. Two selected G/U pairs were found to be required for PSTVd entry into, but not for exit from, the host vascular system. This study identifies critical roles for G/U pairs in the survival of an infectious RNA, and increases understanding of structure-based regulation of replication and trafficking of pathogen and cellular RNAs.

## INTRODUCTION

In addition to its roles in translating genetic information into protein, the discovery of diverse regulatory functions has firmly established RNA as a central player in biology. RNA molecules can fold into a multitude of conformations that often constitute the basis of their function. The major elements of RNA secondary structure can be described as simple loops connecting helical base paired stems. *Cis* Watson–Crick (WC) G/U base pairs, also called G/U wobble pairs, are the most common non-canonical pairs found in RNA. G/U base pairs usually reside in stem regions where they can substitute for classical WC A/U or G/C base pairs due to their comparable thermodynamic stability and near isosteric structure (1). However, their unique geometry and structural flexibility furnish greater potential for RNA-protein and RNA–RNA interaction (2). G/U wobble was first recognized in the context of decoding mRNA codons (3). Additional functions for specific G/U base pairs have since been uncovered, for example, in alanine tRNA synthetase recognition (4–7), group I and group II introns (8–10), hepatitis delta virus ribozyme (11,12), and the human immunodeficiency virus-1 intron splicing silencer (13). G/U base pairs also uniquely form tertiary structure along-groove helix-packing motifs in ribosomal RNA (14–16). However, to our knowledge, a systematic functional analysis of multiple G/U pairs at a whole molecule level has not been performed.

We selected *Potato spindle tuber viroid* (PSTVd) as a model to approach a more comprehensive understanding of G/U pairing status and function. The PSTVd genome is a circular, non-coding RNA of 359 nucleotides that replicates and spreads systemically in infected host plants (17,18). Rolling circle replication occurs in the nucleus where, with the aid of a variant Transcription Factor IIIA, the circular plus-strand genome is transcribed by host RNA polymerase II to generate linear single-stranded (minus strand) concatemers (19). These in turn serve as template for single-stranded (plus strand) concatemers that are subsequently processed to unit length and circularized between positions G95 and G96 by DNA ligase I (20). Circular progeny genomes are then transported out of the nucleus for cell-to-cell and long-distance systemic spread (21–24). Because PSTVd does not encode protein, successful infection of necessity relies on interactions between viroid RNA sequence and/or structure elements and specific host factors, such as proteins and RNAs. These unique biological features make PSTVd an exceptional model to investigate the functional roles of G/U pairs in RNA replication and trafficking.

The PSTVd genome (intermediate strain, PSTVd-I) has been proposed to fold into a rod-shaped structure (referred to here as the canonical structure) (25), which is supported by biochemical and genetic analyses and is thought to be critical for infectivity (26–29). The canonical secondary structure has 17 places where G occurs opposite U in a helix, which we refer to as G/U pairs. However, no atomic-resolution three-dimensional (3D) structure is available, so we do not have confirmation that these 17 G/U pairs actually form G/U base pairs. Moreover, as we show below, G/U and A/U pairs at the ends of helices often do not form WC base pairs. Therefore, one goal of this study was to assess the base pairing status of each of the 17 G/U pairs.

Selective 2′-hydroxyl acylation analyzed by primer extension (SHAPE) (30), combined with RNAstructure software (31), has been used to derive high quality secondary structure predictions for many RNAs, including several viroid genomes (32–35). However, a recent whole molecule SHAPE analysis generated a solution structure for PSTVd-I that contains only 13 of the 17 predicted G/U pairs (36), raising questions about the true nature of PSTVd secondary structure and the ability of whole molecule SHAPE to discern certain structural features.

Here, we report a structural and functional analysis of G/U pairs in PSTVd-I. SHAPE analysis of full-length genomes, and of fragments that collectively span the genome, supported the presence of 14 and 16 of the 17 predicted G/U pairs in the canonical secondary structure, respectively. Using a novel strategy to obtain full-length genome sequences, deep sequencing of progeny populations (quasispecies) derived from wild type PSTVd infection of *Nicotiana benthamiana* revealed that base substitutions at the 17 G/U pairs occurred with a frequency similar to substitutions at canonical WC pairs, and were considerably less frequent than expected by chance. Further, G/U pairs were, on the whole, more likely to be replaced by A/U or G/C pairs than non-canonical base combinations. Mutational analysis of G/U pairs showed that most are essential for PSTVd replication and/or trafficking in *N. benthamiana* plants and protoplasts. We also found that for many positions, A/U and U/U are better able to compensate G/U function than G/C pairs, and that UG could substitute for GU at only two positions without loss of function. Two specific G/U pairs selected for additional analysis were found to be essential for PSTVd trafficking from bundle sheath cells into the vascular system, but not for transit in the opposite direction. In addition, an examination of solved RNA 3D structures showed that G/U pairs in a specific context most often form non-wobble base pairs or no base pair at all. Nevertheless, we observed that PSTVd G/U pairs in this context could have essential functions.

These studies confirm the presence of most G/U pairs in PSTVd, and show that most are important or essential for successful infection. They also suggest that requirements for RNA transit between bundle sheath and phloem cells are unique and directional. Our findings have significant implications for understanding the role of RNA structure in regulating RNA replication and trafficking of both viroid and cellular RNAs.

## MATERIALS AND METHODS

### Plant and protoplast inoculation

*Nicotiana benthamiana* plants were grown in the Biotechnology Support Facility greenhouse at The Ohio State University. The intermediate strain of PSTVd (PSTVd-I) (GenBank accession number NC_002030) was used in these experiments (25). The PSTVd-NB variant used for comparison (GenBank accession number: AJ634596.1) is derived from PSTVd-I and differs by only six nucleotide substitutions (37). Infection was achieved by mechanically inoculating 300 ng of linear plus-strand (+)-PSTVd *in vitro* transcripts onto the upper surface of the first two true leaves of two-week old *N. benthamiana* plants that were previously dusted with carborundum powder. In some experiments, plants were inoculated with circular (+)-PSTVd RNA by needle puncture of stems, petioles, and midveins using a 10 μl syringe (Kloehn #1010, IMI Precision Engineering, Las Vegas, NV, USA). Diethylpyrocarbonate (DEPC) treated water was used for mock inoculation. *Nicotiana benthamiana* protoplasts were prepared and transfected in the presence of polyethylene glycol (PEG) as described (38). Inocula consisted of 6 μg of (+)-PSTVd transcripts mixed with 20 μg of a GFP-encoding plasmid, which served as an indicator of PEG-mediated transformation efficiency.

### RNA preparation

(+)-PSTVd transcripts used to inoculate plants and protoplasts were prepared from pRZ-Int (pRZ6-2-Int) and mutant derivatives. This plasmid, which contains a cDNA copy of PSTVd-I, was a gift of Dr. Robert Owens (39). HindIII-linearized plasmids were used as template for *in vitro* transcription with T7 Megascript (ThermoFisher Scientific, Waltham, MA) according to the manufacturer's instructions. DNase I was used to degrade template DNA, and PSTVd transcripts were purified using the MEGAClear kit (ThermoFisher Scientific). To prepare inoculum for needle puncture, PSTVd transcripts were circularized with T4 RNA ligase according to the method of Beaudry and Perrault (40).

Antisense PSTVd riboprobes used for RNA blot hybridization were prepared and labeled with [α-^32^P]-UTP or digoxigenin (DIG) by *in vitro* transcription using the T7 Maxiscript kit (ThermoFisher Scientific) with SpeI-linearized pInter(–) plasmid (41). Unincorporated UTP was removed using Sephadex G-25 columns (GE Healthcare Life Sciences, Chicago, IL, USA).

### SHAPE experiments

RNA preparation and SHAPE reactions were performed essentially as described (34). Briefly, the circular (+)-PSTVd genome was reverse transcribed from infected plant extracts with two different primers designed to produce monomeric cDNAs beginning at nucleotides 175 or 321. RNAfold (http://rna.tbi.univie.ac.at/cgi-bin/RNAfold.cgi) and Mfold (http://unafold.rna.albany?q=mfold) predicted that monomeric RNAs generated from these cDNAs form similar rod-shaped structures based on minimal free energy. The RT primers contained a T7 RNA polymerase promoter to allow synthesis of full-length genomic RNA, which was dissolved in 0.5× TE, pH 8.0, heated to 95°C for 3 min, and cooled on ice for 5 min. RNA was then incubated at 37°C for 5 min in folding buffer (500 mM Tris–HCl, pH 7.5, 500 mM NaCl) and, following addition of MgCl_2_ to 10 mM, incubated a further 30 min. After folding, a native polyacrylamide gel electrophoresis step was added to ensure homogeneity. The folded genomic RNAs were run on a 5% native gel for 3 h at 45 V in ice cold 0.5× TBE running buffer, with MgCl_2_ (10 mM) added to both the gel and running buffer. To ensure functionality, infectivity was determined by infecting 10 plants with each transcript, followed by RNA extraction and blot analysis to monitor the infection rate. Once transcript homogeneity and infectivity were validated, RNAs were incubated with SHAPE reagent (benzoyl cyanide, BzCN, 600 mM in dimethyl sulfoxide, DMSO) and nucleotide reactivity was assessed by primer extension catalyzed by reverse transcriptase (SuperScript Reverse Transcriptase III, ThermoFisher Scientific). DMSO without BzCN was added to negative control reactions. Experiments were performed in triplicate for each transcript. Electropherograms were analyzed using QuSHAPE (42), and normalized and averaged data were evaluated using RNAstructure (Fold tool, soft thermodynamic constraints) (31). SHAPE analysis of partial PSTVd-I genome sequences using *N*-methylisatoic anhydride (NMIA) as probing chemical was performed in collaboration with the EteRNA project (43).

### Deep sequencing PSTVd quasispecies

Progeny populations obtained from *N. benthamiana* plants inoculated with RNA generated from a cloned wild type PSTVd-I master sequence were profiled using a novel approach that allowed deep sequencing of complete unit-length progeny genomes. RNA samples were obtained from two groups of four plants each and pooled samples were used as biological replicates for deep sequencing. RNA was extracted using RiboZol (VWR Life Science, Radnor, PA, USA) according to the manufacturer's instructions. One pool consisted of RNA obtained from inoculated (local) leaves harvested eight days and ten days post-inoculation (dpi), and another from systemically infected leaves harvested 14 and 21 dpi. Reverse transcription of circular genomes was conducted with a PSTVd-specific primer and SuperScript Reverse Transcriptase III (ThermoFisher Scientific), which has strand-displacement activity. Strand displacement permitted reverse transcription of greater than unit-length cDNAs, which were then used for PCR amplification. A pair of primers was designed to amplify unit-length PSTVd cDNA (359 bp) between the forward and reverse primers. Adaptors were added using NEBNext Ultra Directional RNA Library Kit for Illumina (New England Biolabs, Ipswich, MA), and PCR with primers containing barcode and sequencing primer binding site sequences was then performed. Final products had adaptors and binding sites for sequencing primers on both ends and a barcode on one end.

Sequencing was performed using the MiSeq platform (Illumina, San Diego, CA, USA). This technology sequences greater than 250 bp from each end, so that together paired end sequences spanned entire unit-length PSTVd cDNAs. For this analysis, only sequences longer than 250 nt were used. Raw sequencing data was processed using a Python-based pipeline and analyzed using the Mothur software package as described (44). Briefly, the ‘make.contigs’ function was used to build contigs after removing adaptor sequences. In addition, primer sequences (first 20 and last 19 bases) were removed. The ‘screen.seqs’ function was used to remove sequences with ambiguous bases and sequences that were longer than desired length. The ‘unique.seqs’ and ‘count.seqs’ methods were used to identify unique sequences in each dataset. The 17 G/U sites were taken from sequences in each of the libraries and recorded into 17 individual output files. Unique base combinations at these G/U sites were recorded and listed.

### Construction of G/U mutants

PSTVd mutants were generated by site-directed mutagenesis using a plasmid pRZ6-2 as template (39). Methods for site-directed mutagenesis have been previously described (45). All mutant constructs were sequenced to verify the introduced mutations.

### RNA extraction and RNA blots

Inoculated (local) and systemically infected leaves were sampled for RNA extraction 10 and 28 dpi, respectively. Transfected protoplasts were collected for RNA extraction at 3 dpi. RNAzol reagent (Sigma-Aldrich, St. Louis, MO, USA) was used to extract total RNA from PSTVd- and mock-inoculated *N. benthamiana* plants and protoplasts following instructions from the manufacturer. RNA blotting was performed essentially as described (27).

### Sequencing PSTVd progeny

RNA was obtained from systemically infected leaves using RNAzol (Sigma-Aldrich). Protocols used to synthesize cDNAs have been described (41). Briefly, SuperScript III Reverse Transcriptase (ThermoFisher Scientific) was used to prepare the first cDNA strand with two primers: PSTVd1S (5′-AGGAACCAACTGCGGTTCCA-3′) or PSTVd95S (5′-GGGGATCCCTGAAGCGCTCC-3′), to cover the entire genome. The second cDNA strand was synthesized using the HotStart-IT Taq DNA Polymerase PCR system (ThermoFisher Scientific) with two primer pairs: PSTVd1F (CGGAACTAAACTCGTGGTTCCT) and PSTVd1S; or PSTVd95F (GGGGAAACCTGGAGCGA) and PSTVd95S, following the manufacturer's instructions. PCR cycling conditions were as follows: 40 cycles at 95°C for 30 s, 55.4°C for 30 s, and 72°C for 40 s, with a final extension step at 72°C for 10 min. PCR products were analyzed by electrophoresis on a 1.5% agarose gel and cloned into a pGEM-T vector (Promega Life Sciences, Madison, WI, USA) for sequencing.

### RNA degradation assay

The relative stabilities of wild type PSTVd and mutant G76A and G156A *in vitro* transcripts were assessed in buffer or *N. benthamiana* leaf extracts as previously described (46). Briefly, extracts were prepared by grinding young leaf tissue (100 mg) in 1 ml buffer (20 mM Tris–HCl, pH 7.5, 150 mM NaCl, 10 mM phenylmethanesulfonyl fluoride) (PMSF, Sigma-Aldrich). The supernatant was collected following centrifugation to remove debris. RNA degradation assays were performed by mixing wild type and mutant transcripts (10 ng) with the supernatant (90 μl), or buffer only as a control, at 28°C. Samples were taken at 0, 15, 30, 60, 120 and 180 min and immediately frozen with liquid nitrogen. RNA blot analysis was performed and signals were quantified using Quantity One software (Bio-Rad, Hercules, CA, USA). Degradation curves were prepared using the quantified data.

### *In situ* hybridization

Samples were prepared as previously described (37,47,48), with slight modifications. The main differences were that samples were fixed in 3.7% FAA solution (50% ethanol/3.7% formaldehyde/5% acetic acid) overnight at 4°C, and hybridization with DIG-labeled PSTVd antisense riboprobes was carried out at 55°C overnight. Whole-mount *in situ* hybridization was performed following the protocol described by Traas (2008) (49).

## RESULTS

### G/U base combinations in RNA 3D structures according to secondary structure context

PSTVd has a known sequence and predicted secondary structure, as we will describe below, but no atomic-resolution 3D structure, so we must rely on inferences about the detailed structure of G/U and other base pairs in the molecule. (Throughout, G/U is used to indicate either GU or UG pairs, and this notation also applies to other types of pairs.) We used 3D structures of other RNA molecules to learn how often it happens that G and U nucleotides that are opposite each other in the secondary structure actually form G/U wobble base pairs using their WC edges. We refer to these as cWW base pairs, short for *cis* Watson–Crick/Watson–Crick in the Leontis/Westhof nomenclature (50). We investigate three secondary structure contexts: Case 0, Case 1 and Case 2.

**Figure 1.**
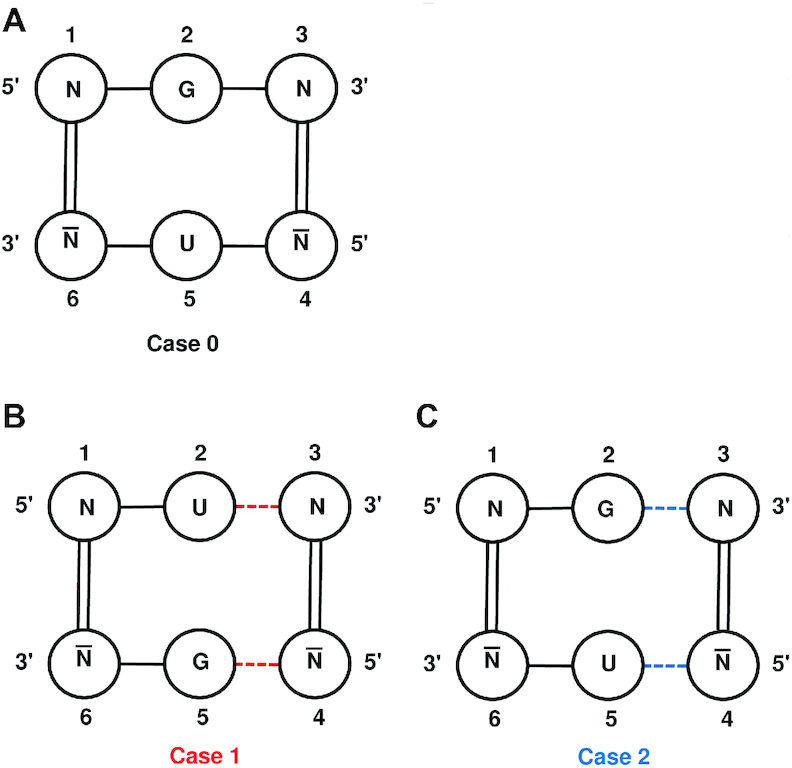
G/U pairs in different secondary structure contexts. (**A**) Case 0, G opposite U, sandwiched between canonical A/U or G/C WC base pairs. The abbreviation N stands for any RNA base, and N-bar for its WC complementary base. (**B**) Case 1, positions 1 and 6 are WC paired, representing the end of a helix, U is at the 3′ end of one helix strand, G is at the 5′ end of the other helix strand, positions 3 and 4 are WC base paired, and positions 3 and 4 are separated from 2 and 5 by a single-stranded region (red dashed lines) so that they enclose an internal loop. (**C**) Case 2, like Case 1, except that G is at the 3′ end of the helix strand and U is at the 5′ end, positions 3 and 4 are WC base paired, and positions 3 and 4 are separated from 2 and 5 by a single-stranded region (blue dashed lines) so that they enclose an internal loop.

Case 0 is when G and U are opposite each other within the secondary structure, sandwiched between canonical G/C or A/U WC base pairs, as shown in Figure 1A. The program FR3D (Find RNA 3D) (51) was used to find six-nucleotide motifs with positions 1 and 6 making a canonical WC pair, positions 3 and 4 doing the same, position 2 being G, position 5 being U, positions 1, 2, 3 being sequentially adjacent, and positions 4, 5, 6 being sequentially adjacent. We searched a representative set of 1146 RNA-containing 3D structures of all sizes, release 3.48, resolution threshold 3.0 Å (http://rna.bgsu.edu/rna3dhub/nrlist/release/3.48/3.0A) and found 387 instances of the motif, of which 374 (96.6%) had an annotated cWW base pair (or near base pair) between G in position 2 and U in position 5. The exceptions were one instance of a near *cis* Hoogsteen/Watson–Crick interaction and 12 instances with a near or true *cis* Watson–Crick/Sugar edge base pair between positions 2 and 5; this change in annotation requires just a small rotation of the U, and these dynamic molecules can switch between cWW and cWS fairly easily. For comparison, in the same context, G/C forms a cWW base pair in 99.66% of the instances, A/U forms a cWW base pair in 99.50% of the instances, and U/U forms a cWW base pair in 95.2% of the instances (Table 1). Thus, all of these base combinations can be expected to form a cWW base pair in the interior of a helix.

**Table 1.** Base combinations in RNA 3D structures according to secondary structure context

	Case 0			Case 1			Case 2		
	Instances	% cWW	% no bp	Instances	% cWW	% no bp	Instances	% cWW	% no bp
GC	3237	99.66%	0.15%	673	97.5%	1.9%	602	96.5%	2.5%
AU	1804	99.50%	0%	277	74.3%	18.1%	261	86.6%	8.4%
GU	387	96.6%	0%	239	86.2%	9.6%	85	22.4%	51.8%
UU	21	95.2%	0%	259	82.6%	16.2%	Same as case 1 by symmetry		

Results of RNA 3D structure search for base combinations GC, AU, GU, and UU found in release 3.48 of the representative sets in the three secondary structure contexts Case 0 (helix interior), Case 1 (A or G on 5′ end of a helix strand), and Case 2 (U or C on 3′ end of a helix strand). Counts of total instances are given, then the percentage of instances in which the bases in positions 2 and 5 make a cWW or near cWW base pair, and the percentage of instances in which they make no base pair or near base pair of any type. UU Case 2 is the same as UU Case 1, so only one set of numbers is given.

Case 1 is when U and G occur opposite one another at the end of a helix, next to an internal loop, with the U at the 3′ end of one helix strand, and the G at the 5′ end, as shown in Figure 1B. We used FR3D to search for instances where the nucleotides at positions 1 and 6 and 3 and 4 form canonical WC base pairs, 2 is U, 5 is G, positions 1, 2, 3 are in increasing nucleotide order and 1 and 2 are adjacent, and positions 4, 5, 6 are in increasing nucleotide order and 5 and 6 are adjacent, and any nucleotides between positions 2 and 3 and between 4 and 5 do not make WC base pairs, and so are interior to an internal loop. FR3D found 239 instances of this motif, and in 206 cases (86.2%), the G and U made a cWW base pair or near base pair. In 10 cases (4.2%), the G and U make a cWS base pair or near base pair, and in 23 cases (9.6%), they make no base pair. Thus, a large majority of Case 1 G/U pairs form a cWW base pair. For comparison, replacing U/G with C/G in this context, 97.47% of instances have a cWW base pair, for U/A, 74.3% make a cWW base pair and 18.1% make no base pair, and for U/U, 82.6% make a cWW base pair and 16.2% make no base pair (Table 1). Thus, C/G reliably makes a cWW base pair in this context, U/G next, then U/U, and then U/A.

Case 2 is the same as Case 1, except with the positions of G and U reversed, so G is at the 3′ end of one helix strand and U is at the 5′ end, as shown in Figure 1C. FR3D found just 85 instances of this motif, many fewer than Case 1, with just 19 instances (22.4%) making a cWW base pair or near base pair, 15 instances (17.6%) of a G/U cWS base pair or near base pair, 7 instances (8.1%) of a G/U trans Sugar/Hoogsteen base pair or near base pair and 44 instances (51.8%) of no base pair at all. Thus, in just over half of the observed instances in high-resolution RNA 3D structures, G/U in Case 2 does not make a base pair. For comparison, replacing G/U with G/C in this context, 96.5% make a cWW base pair, and for A/U, 86.6% make a cWW base pair. U/U is the same in Case 1 and Case 2 due to symmetry (Table 1). Thus, G/C reliably makes a cWW base pair in this context, A/U next and G/U forms a cWW base pair at a very low rate.

In summary, base combinations GC, AU, GU and UU almost always make cWW base pairs in the interior of a helix (Case 0). At the end of a helix in Case 1 context, GU is very likely to make a cWW base pair. GU at the end of a helix in Case 2 context is uncommon and when it occurs, it only makes a cWW base pair in 22% of the instances, with no base pair at all in 52% of the instances. These observations are consistent with the contributions of G/U pairs to overall helix free energy based on nearest-neighbor parameters (https://rna.urmc.rochester.edu/NNDB/turner04/gu-references.html) (52). As shown in Table 2, the contribution of Case 2 context G/U is weaker than Case 1, and thus Case 2 G/U combinations are less likely to form base pairs.

**Table 2.** Free energy parameters for G/U pairs

	5′U-3′G	5′G-3′U
	(akin to Case 1)	(akin to Case 2)
5′G-C3′	−2.5	−1.5
5′C-G3′	−2.1	−1.4
5′A-U3′	−1.4	−0.6
5′U-A3′	−1.3	−1.0

General contributions of G/U pairs to overall helix free energy (Δ*G*°_37_ kcal/mol) with respect to adjacent WC base pairs indicated to the left. Values applicable to Case 1 and Case 2 contexts are indicated.

### SHAPE confirms most G/U pairs in PSTVd

**Figure 2.**
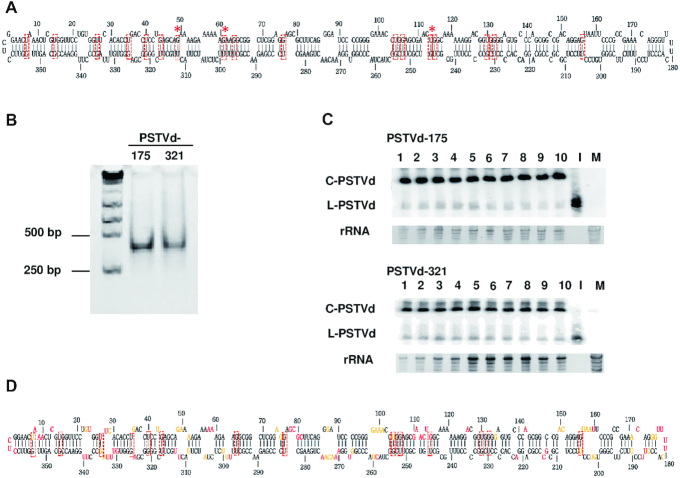
Analysis of PSTVd G/U pairs by whole molecule SHAPE. (**A**) The canonical secondary structure of PSTVd containing 17 G/U pairs (red boxes) is shown. Red asterisks indicate G/U pairs not supported by whole molecule SHAPE (see 1D). (**B**) Folded, full-length PSTVd *in vitro* transcripts prepared for SHAPE, beginning at nucleotide positions 175 or 321, were tested for homogeneity by native polyacrylamide gel electrophoresis. A gel stained with ethidium bromide is shown. The positions of 250 bp and 500 bp size markers are indicated. (**C**) The infectivity of PSTVd-175 and PSTVd-321 transcripts was tested by inoculating 10 *N. benthamiana* plants with each RNA. Blots of RNA prepared from systemically infected leaves hybridized with a PSTVd-specific probe are shown. No signal was observed in RNA preparations from mock-inoculated plants (M). A full-length linear PSTVd *in vitro* transcript served as inoculum (I). Circular (C) and linear (L) forms of PSTVd RNAs are indicated. rRNA stained with ethidium bromide was a loading control. (**D**) The PSTVd secondary structure obtained by whole molecule SHAPE and folded with RNAstructure software is shown. Experiments were performed in triplicate with each RNA, and the averaged and similar data were used to derive the structure. The 14 G/U pairs predicted by this analysis are indicated by red boxes. SHAPE reactivity is indicated by nucleotide color: red = high reactivity (>0.85), orange = intermediate (0.40–0.85), black = low (0–0.40).

The canonical secondary structure of PSTVd-I (intermediate strain, hereafter PSTVd) is believed to contain 17 G/U juxtapositions (Figure 2A). To confirm they are base paired, two full-length PSTVd RNA strands, beginning at nucleotide positions 175 and 321, were generated *in vitro* and used for whole molecule SHAPE (30,34). These RNAs formed similar homogeneous structures in solution as judged by native polyacrylamide gel electrophoresis (Figure 2B). To verify their functionality, infectivity was assessed by inoculating 10 *N. benthamiana* plants with each RNA. As PSTVd generates asymptomatic infections in this host, infectivity was confirmed by blot analysis of RNA isolated from upper, non-inoculated leaves (systemically infected leaves) using a PSTVd specific probe. Both RNAs achieved a 100% systemic infection rate (Figure 2C). SHAPE experiments were then performed in triplicate with each of the RNAs. Electropherograms were analyzed using QuSHAPE (42), and the normalized and averaged data were evaluated using RNAstructure software (31).

The most stable predicted PSTVd secondary structure, constructed using the averaged and similar SHAPE reactivity data obtained from the 175 and 321 genome strands, is presented in Figure 2D. Following whole molecule analysis, 14 of the 17 G/U pairs in the canonical structure showed relatively low reactivity to benzoyl cyanide (BzCN) and were predicted to be base paired (Table 3). Reactivities for all PSTVd nucleotides are listed in Supplementary Table S1. G/U pairs at nucleotide positions 49:312, 61:299 and 114:246 in the canonical structure were not supported by this analysis. Two of these, 49:312 and 114:246, are in Case 2 context, while 61:299 is Case 0. Our structure largely agrees with an earlier BzCN-derived whole molecule SHAPE study of PSTVd-I that supported 13 of the 17 G/U pairs (36), lacking one at position 115:245 in our structure. A more recent study used *N*-methylisatoic anhydride (NMIA) and 2-methylnicotininc acid imidazolide (NAI) to perform whole molecule SHAPE of the PSTVd-NB variant, which is derived from PSTVd-I and differs by only six nucleotide substitutions (53). Interestingly, one of these substitutions converts the G/U pair at 44:317 in PSTVd-I to a G/C pair. Apart from this, the NMIA-derived structure also did not support G/U pairs at positions 49:312, 61:299 and 114:246. A similar outcome was obtained with NIA, except a G/U pair was observed at 61:299. Thus, despite the use of three different SHAPE probing chemicals, there is remarkable agreement between the four structures. All four did not discern G/U base pairs at positions 49:312 and 114:246 (both Case 2 context), while three did not detect G/U pair 61:299 (Case 0). A comparison of these PSTVd-I and PSTVd-NB whole molecule SHAPE structures is presented in Supplementary Figure S1.

**Table 3.** Normalized reactivities for PSTVd G/U pairs determined by whole molecule SHAPE

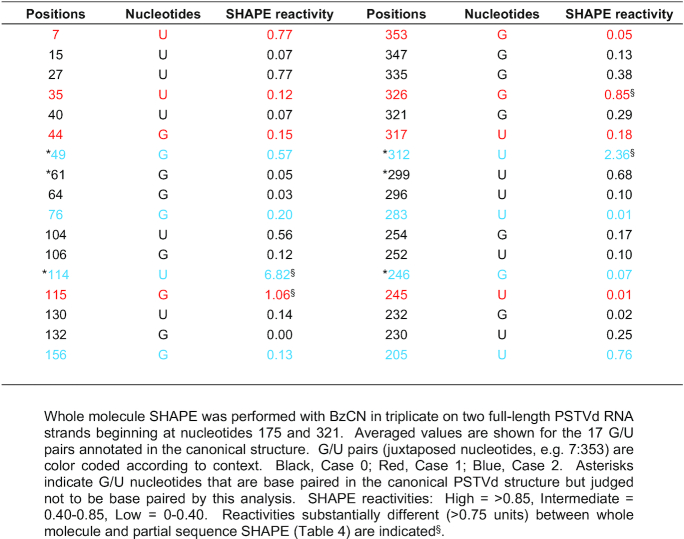

In a complementary approach, SHAPE was performed with NMIA on nine fragments that collectively encompass most of the PSTVd genome (Supplementary Figure S2; Supplementary Table S2). In this study, done in collaboration with the EteRNA project (43), 16 of the 17 G/U pairs in the canonical structure displayed low SHAPE reactivity and were likely base paired (Table 4). The reactivities of nucleotides at the remaining position (61:299) could not be determined due to its location at the junction between two fragments. Overall, reactivities obtained from whole molecule SHAPE using BzCN and partial fragment SHAPE using NMIA were similar, although in general values from partial fragments were somewhat lower (Supplementary Tables S1 and S2). However, large differences (approaching one unit) were not common, and occurred at fewer than 10% of total nucleotides. The sizeable differences noted at nucleotides involved in G/U pairs 35:326, 49:312, 114:246 and 115:245 (Tables 3 and 4) may be due to the use of different SHAPE chemicals and/or differences in folding between whole genomes and fragments. In any event, SHAPE using PSTVd genome fragments further supported annotation of the 17 G/U pairs in the canonical structure.

**Table 4.** Normalized reactivities for PSTVd G/U pairs from fragments spanning the genome

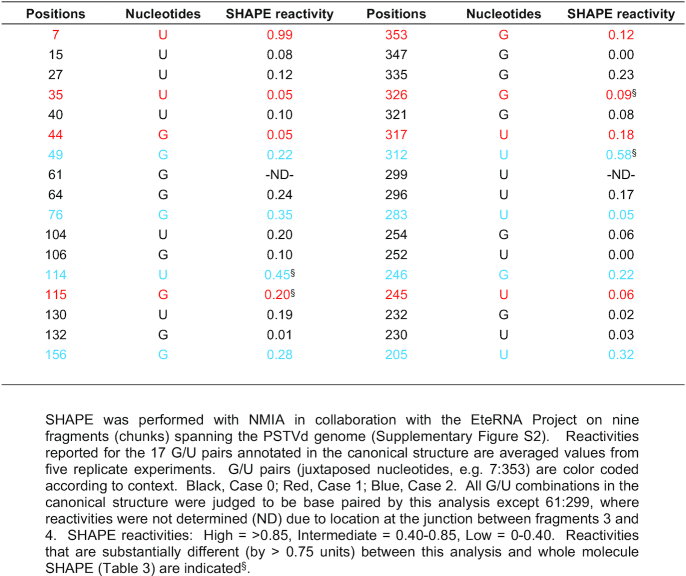

Overall, the SHAPE results suggest that 14 of the G/U pairs form stable structures under different experimental conditions, while the remaining three show variable results that may be indicative of weaker, more transient base pairing. On this basis, we concluded that further study of all 17 PSTVd G/U pairs was warranted.

### Deep sequencing PSTVd progeny derived from a master sequence reveals conservation of G/U pairs

**Figure 3.**
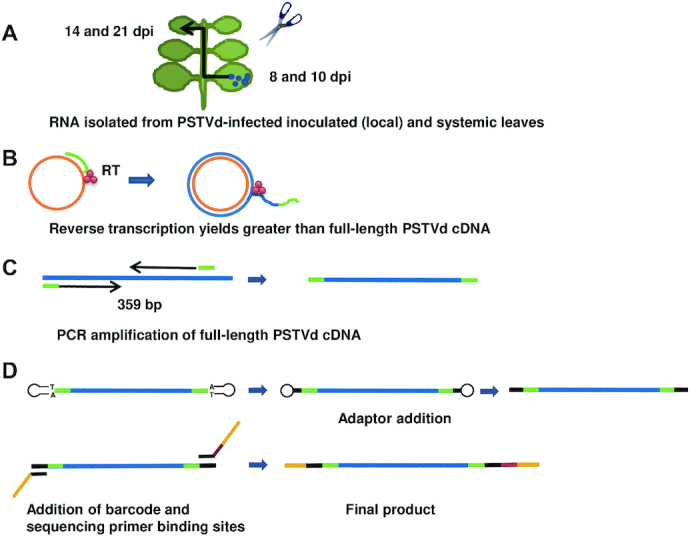
Strategy for deep sequencing full-length PSTVd progeny genomes. (**A**) RNA was extracted from pooled leaves harvested 8- and 10 dpi for local (inoculated) leaf libraries, and 14 and 21 dpi for systemic leaf libraries. (**B**) RT-PCR was conducted with a sequence specific primer (green) and reverse transcriptase (RT) with strand displacement activity, allowing transcription of greater than full-length cDNA (blue) from circular (+)-PSTVd RNA (orange). (**C**) Greater than full-length cDNA was used for PCR amplification. A primer pair (green) was designed to amplify full-length PSTVd cDNA (359 bp). (**D**) Adaptors (black), barcode (brown), and sequencing primer binding sites (yellow) were added by PCR. The final products contained sequencing primer binding sites and adaptors on both ends and a barcode on one end.

To assess the type and frequency of natural sequence variation at G/U pairs that might occur during infection, *N. benthamiana* plants were inoculated with *in vitro* transcripts derived from cloned PSTVd. Progeny genome populations, which can be considered quasispecies, were sequenced to identify variants. Deep sequencing was accomplished using a novel approach that takes advantage of a reverse transcriptase capable of strand displacement to prepare greater than full-length cDNA copies from circular (+)-strand PSTVd RNA genomes, the predominant form found in infected cells. Following amplification of unit-length cDNA by PCR, the MiSeq platform (Illumina) was used for paired-end sequencing. This technology allows more than 250 bp to be sequenced from each end, so that paired-end sequences spanned complete PSTVd genomes embedded within the original cDNAs. The procedure is outlined in Figure 3 and further described in Materials and Methods.

In an effort to capture the greatest number of possible variants, extracts were prepared from pooled inoculated (local) leaves harvested at 8 and 10 dpi. Similarly, extracts were prepared from pooled systemically infected leaves collected at 14 and 21 dpi. RNA isolated from the pools was used to prepare local (L) and systemic (S) deep sequencing libraries. Two independent experiments were conducted, and the four libraries (L-1 and S-1, L-2 and S-2) generated more than 2.2 × 10^6^, 2.5 × 10^6^, 1.5 × 10^6^ and 2.5 × 10^6^ complete PSTVd sequences without insertions or deletions, respectively. From these more than 4.9 × 10^4^, 6.0 × 10^4^, 1.1 × 10^5^ and 1.3 × 10^4^ unique sequences were identified. To minimize errors resulting from library preparation, sequences with fewer than 30 reads per million were eliminated, and of the remaining reads ∼89% were wild type PSTVd and ∼11% were variants. Variants with more than one substitution were rare (<3%), and molecules with two substitutions at the same base pair, e.g. GU to UG, were not observed. Nevertheless, to minimize the possibility that one mutation might compensate for another, only variant sequences with one base substitution were used for further analysis. A total of 545 (L-1), 567 (S-1), 262 (L-2) and 726 (S-2) sequences met these criteria. In addition, as previous studies have shown that non-functional variants can be maintained by wild type genomes in plant virus populations (54), only unique sequences shared by cognate local and systemic leaf libraries, which were considered more likely to retain both replication and trafficking functions, were included in the sequence pool used to analyze variation at G/U pairs, leaving 346 L-1+S-1 and 88 L-2+S-2 sequences.

A summary of the types of substitutions observed at G/U pairs in progeny genomes is presented in Table 5. Libraries from systemically infected leaves (S-1 and S-2) generally exhibited a larger number of variant types than those prepared from inoculated local leaves (L-1 and L-2), likely due to the longer time mutations were allowed to accumulate. However, reflecting their stochastic nature, specific substitutions were not always observed in replicate libraries (i.e. L-1 and L-2; S-1 and S-2). Nevertheless, even when only mutations shared by cognate local and systemic libraries (i.e. L-1 and S-1 or L-2 and S-2) are considered, sequence variation was evident at all G/U pairs, except one at position 106:252.

**Table 5.** Variants for each PSTVd G/U pair in quasispecies

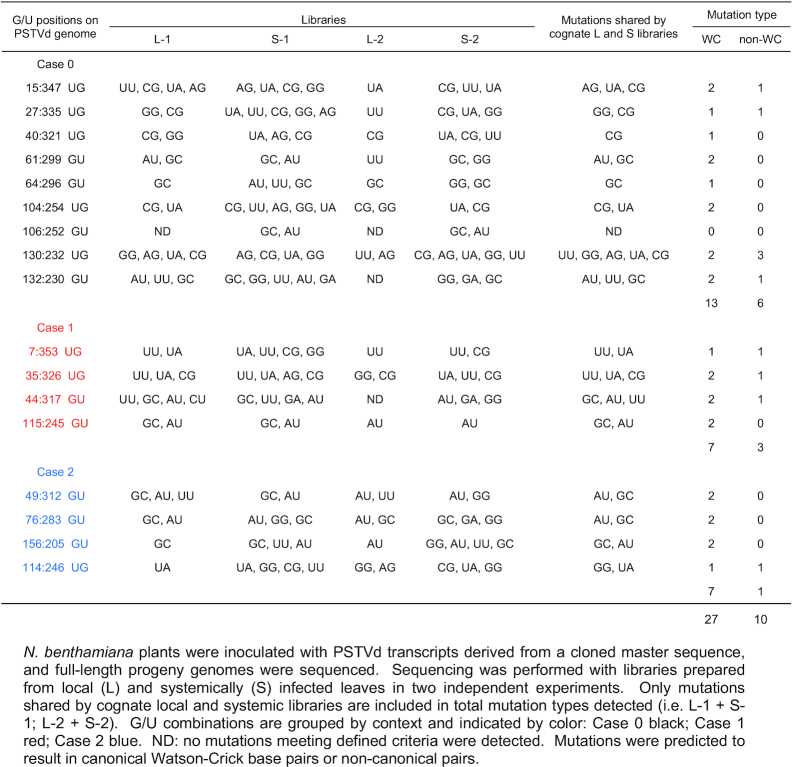

Consistent with a previous report (15), substitution of G/U pairs with G/C or A/U pairs proved to be the dominant type of substitution shared in local and systemic libraries (27 of 37) (Table 5). Substitution by non-canonical base pairs was less common (10 of 37), even though the random probability of a non-WC substitution is greater by 2:1. When considered by context, the nine Case 0 and four Case 1 G/U pairs were about twice as likely to be replaced by WC pairs than non-WC pairs (13:6 and 7:3, respectively). However, substitutions at the four Case 2 G/U pairs nearly always resulted in WC pairs (7:1). Again, no G/U to U/G substitutions were observed.

Three types of potential non-canonical base pair substitutions were shared in cognate L and S libraries: G/U to U/U at five positions (7:353, 35:256, 44:317, 130:232 and 132:230), G/U to G/G at three positions (27:235, 114:246 and 130:232), and G/U to A/G at two positions (15:347 and 130:232). Local and systemic libraries did not share a G/U to C/U substitution, which has the same nominal probability as other non-canonical types. Only one GU to CU substitution was found in one local leaf library (L1) at position 44:317 (Table 5).

We also assessed variation at each individual nucleotide in PSTVd quasispecies, using unique sequences with reads greater than 30 per million and with only one base substitution (∼150,000 reads from the four libraries). Interestingly, substitutions affecting nucleotides in G/C or A/U pairs, and G/U pairs, were under-represented to a similar extent (Table 6). Both types were considerably less frequent than expected by chance (observed/theoretical = 0.574 and 0.546, respectively), suggesting substitutions that disrupt canonical WC or G/U pairs are more likely to have an adverse impact on PSTVd infectivity. Interestingly, G/U combinations in Case 1 and Case 2 contexts, which reside at the ends of base-paired stems and serve to close loops, were least likely to suffer substitutions (observed/theoretical = 0.417 and 0.358, respectively). By contrast, substitutions in loops occurred twice as often as expected (observed/theoretical = 2.012).

**Table 6.** Comparison of nucleotide substitution frequencies in PSTVd quasispecies

			G/U			
	A/U or G/C	Loop	Total G/U	Case 0	Case 1	Case 2
Theoretical	0.607	0.298	0.095	0.050	0.022	0.022
Observed	0.349	0.600	0.052	0.034	0.009	0.008
Observed/theoretical	0.574	2.012	0.546	0.688	0.417	0.358

The proportion of nucleotides involved in A/U or G/C base pairs, G/U pairs, and loops in the PSTVd secondary structure (Theoretical) are compared with the proportion of substitutions detected at these nucleotides (Observed) in the sequenced population derived from a cloned Master sequence. Substitution frequencies for G/U nucleotides in different contexts (Case 0, Case 1 and Case 2) are also indicated.

In summary, we observed that while mutations can occur at almost all PSTVd G/U pairs in natural populations, most often they were replaced by A/U or G/C pairs. In addition, like canonical WC base pairs, nucleotides in G/U pairs were less likely to be replaced than nucleotides in loop regions. Thus, our analysis of PSTVd quasispecies provides additional support for the presence and importance of G/U base pairs in the PSTVd secondary structure.

### Functional analysis identifies PSTVd G/U pairs essential for replication and systemic spread

To assess their biological significance, the 17 G/U pairs in the canonical PSTVd structure were subjected to mutational analysis. G/U and classical Watson–Crick base pairs share similar structures, but the former have greater conformational flexibility. Thus, we replaced each G/U pair with A/U and G/C, with A/U pairs being more flexible than G/C pairs (two versus three hydrogen bonds). In addition, each G/U pair was substituted with U/U, a non-canonical pair that has a different structure and greater flexibility than G/U (55,56). The hydrogen bonding properties of these cWW base pairs are illustrated in Supplementary Figure S3. Finally, because GU and UG pairs are not self-isosteric (15), G/U pairs were replaced with U/G by double mutation to assess the importance of orientation. Each mutant was tested for infectivity on ten *N. benthamiana* plants. As PSTVd causes asymptomatic infections in this host, RNA extracts from harvested leaves were analyzed by blot hybridization with a PSTVd-specific probe. RNA samples were obtained 10 dpi from inoculated (local) leaves, where positive signals for PSTVd were taken to indicate successful replication. It should be noted that in local leaf samples, circular progeny genomes are easily distinguished from linear residual inoculum due to their much slower mobility in polyacrylamide gels (39). RNA was isolated from systemically infected leaves at 28 dpi, where positive signals potentially indicate successful replication and trafficking. To confirm successful infection, the maintenance of each introduced mutation was evaluated by sequencing one or two full-length PSTVd progeny cloned from extracts of pooled systemically infected leaves. In control inoculations performed with each experiment, a 100% systemic infection rate (10/10 plants) was invariably achieved with wild type PSTVd (positive control), whereas no evidence of infection was observed in mock-inoculated plants (negative control).

#### PSTVd G/U pairs involved in replication

**Figure 4.**
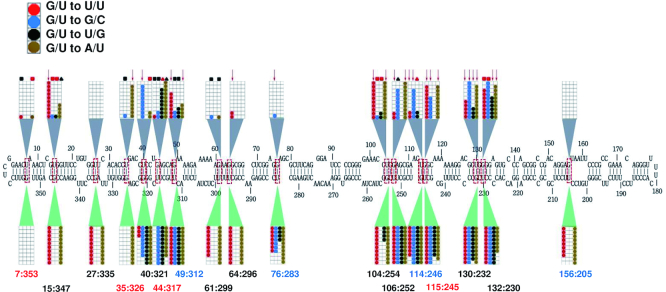
Replication and trafficking rates for PSTVd G/U mutants. The nucleotide coordinates for each G/U pair is given below the diagram of the canonical PSTVd secondary structure, with sequence context indicated by color: Case 0 (black), Case 1 (red), Case 2 (blue). Ten *N. benthamiana* plants were inoculated with each mutant and RNA from inoculated (local) and systemically infected leaves was evaluated for the presence of PSTVd by RNA blot analysis. For each mutation type, 10 boxes within a grid represent 10 inoculated plants. Lower grids show data for local inoculated leaves, where positive signals indicate replication. Upper grids show data for systemically infected leaves, where positive signals indicate potential systemic trafficking. Colored circles represent infected plants. Four different mutants were generated for each pair: G/U to U/U (red circles); G/U to G/C (blue), G/U to U/G (black) and G/U to A/U (brown). Symbols above the diagram represent results of progeny sequencing from systemically infected leaves. Red arrow: retained the introduced mutation without acquiring new mutations. Black square: introduced mutation(s) reverted to wild type. Black triangle: introduced mutation retained and new mutations acquired. Red square: introduced mutation(s) reverted to wild type and new mutations acquired. Red triangle: for G/U to U/G, retained one of the two introduced mutations and acquired new mutations.

The outcome of infectivity experiments with all 68 mutants is summarized in Figure 4. The majority of the mutants (49 of 68) were capable of replicating in inoculated leaves (indicated by grids below the canonical PSTVd structure in Figure 4), and only one G/U pair (7:353) did not replicate with any of the introduced mutations. Conversely, at ten G/U pairs (40:321, 44:317, 49:312, 76:283, 104:254, 106:252, 114:246, 115:245, 130:232 and 132:230) three or more mutation types were able to replicate in inoculated leaves. These more permissive G/U pairs were mainly clustered in two regions of the secondary structure, with three between 40:321 to 49:312 and six between 104:254 and 132:230, with an additional site (76:283) between the two clusters. Eight of these G/U pairs are next to or near another G/U pair in the same WC base-paired stem (44:317/49:312, 104:254/106:252, 114:246/115:245 and 130:232/132:230), suggesting that mutations in one pair might be mitigated to some extent by the other. At the remaining six G/U pairs, only mutations to A/U (35:326) or A/U and U/U (15:347, 27:235, 61:299, 64:296 and 156:205) were tolerated. Only two of these pairs (61:299/64:296) are located in the same base-paired stem.

While replication was not detected for any of the tested mutation types at 7:353, G/U to A/U substitutions at any of the other 16 G/U pairs did not significantly impair replication (Figure 4). Surprisingly, while one might expect mutations to WC base pairs to have the least impact on phenotype, we found that U/U mutants were functional in nearly as many sites as A/U mutants. Most U/U mutants (14 of 17) were able to replicate, and at the six G/U pairs noted above U/U and/or A/U were the only replication-competent mutants. By comparison, nine of 17 G/U to G/C and ten of 17 G/U to U/G mutants could replicate, and these were always found together at permissive G/U pairs that allowed replication of multiple mutation types, including A/U and U/U. No clear correlation between G/U context (Case 0, 1 or 2) and replication of a particular mutation type was observed.

In summary, we observed that one G/U pair (7:353, discussed further below) is essential for PSTVd replication. In addition, six G/U pairs (15:347, 27:335, 35:326, 61:299, 64:296 and 156:205) tolerated substitutions resulting in A/U or A/U and U/U pairs, but not more rigid G/C pairs or non-isosteric G/U to U/G, suggesting that a particular conformation and flexibility at these locations might have some role in replication.

#### PSTVd G/U pairs essential for systemic trafficking

A majority of the mutants (43 of 68) also appeared to be capable of systemic spread (indicated in grids above the PSTVd sequence in Figure 4). Based on RNA blot analysis, only two G/U pairs (27:335 and 156:205) did not tolerate any of the introduced mutations. However, upon sequencing progeny from systemically infected leaves it was found that original mutant genomes were maintained intact in only 22 of 68 cases (i.e. retained the introduced mutation and did not acquire new ones, indicated by red arrows in Figure 4) (Supplementary Table S3). In five of these cases systemic infection rate was very low, with fewer than 4/10 plants infected (64:296 GU to UU, 76:283 GU to GC, 106:252 GU to UU, 114:246 UG to UU, 130:232 UG to GU). Thus, 22 mutants were genetically stable and capable of systemic spread, although five of these were impaired in this respect.

Of the remaining 21 mutants that were not genetically stable, 15 exhibited a poor systemic infection rate (fewer than 4/10 plants infected). Further, progeny recovered from systemic leaves had either reverted to wild type (8 cases, indicated by black squares in Figure 4), retained the introduced mutation but introduced new and possibly compensatory mutations (3 cases, indicated by black triangles), or the introduced mutation reverted to wild type and new mutations were acquired (8 cases, indicated by red squares). In two G/U to U/G double mutants (44:317 and 114:246), progeny retained only one of the original mutations and acquired new mutations at other sites (indicated by red triangles). Surprisingly, at three locations (15:347, 49:312 and 104:254), introduced GU to UG double mutations reverted to wild type (Supplementary Table S3). Similar double mutations at the same base pair were not observed in natural variants.

The 22 mutants that were genetically stable and capable of at least limited systemic trafficking were distributed among 12 of the 17 PSTVd G/U pairs. However, distribution was not even. As was noted for replication, G/U pairs present in a base-paired stem with another G/U pair were more permissive of mutation. Seven of these G/U pairs (49:312, 104:254, 106:252, 114:246, 115:245, 130:232 and 132:230) accounted for 17 of the 22 mutants capable of systemic spread, and all tolerated at least two mutation types. Five additional G/U pairs allowed spread of a single mutation type (15:347, 35:326, 40:321, 64:296, 76:283). However, as noted below, several mutants exhibited impaired systemic spread.

Analysis of mutations by type again revealed that G/U to U/U mutants were as likely or more likely to be viable than mutants having a canonical WC base pair in place of G/U. Of the U/U mutants tested, eight of 17 were viable, although three were impaired for systemic spread (indicated by an asterisk*) (15:347, 49:312, 64:296*, 104:254, 106:252*, 114:246*, 115:245 and 130:232). By comparison, seven of 17 G/U to A/U mutants (35:326, 49:312, 104:254, 106:252, 115:245, 130:232, and 132:230) and five of 17 G/U to G/C mutants proved viable, with one severely impaired for systemic spread (40:231, 76:283*, 114:246, 115:245 and 130:232). By contrast, only two G/U to U/G mutants were viable, and one of these was impaired for systemic spread (130:232* and 132:230). No correlation between mutation type and context of G/U pairs was evident.

In summary, applying stringent criteria of replication, efficient systemic trafficking, and maintenance of intact mutant genomes, 10 of the 17 G/U pairs could tolerate at least one type of mutation. Of the remaining seven pairs, mutations at one (7:353) abolished replication, mutations at four (27:335, 44:317, 61:299 and 156:205) blocked systemic spread, and mutations at two additional G/U pairs (64:296 and 76:283) greatly impaired systemic spread. Thus, from this analysis of single mutants (and GU to UG double mutants), these seven G/U pairs appear to be essential for infectivity.

### UG pair 7:353 is essential for PSTVd replication

The 7:353 UG pair lies within a region known to bind DNA-dependent RNA polymerase II (Pol II) and its co-factor, a seven zinc finger splice variant of Transcription Factor IIIA (TFIIIA-7ZF) (19,57), which together transcribe (+)-PSTVd RNA to initiate the replication process. SHAPE reactivity for this pair was 0.77/0.05 (whole molecule SHAPE) and 0.99/0.12 (genome fragment SHAPE), supporting a possible base pair. While UU and UA variants were identified from deep sequencing (Table 5), these and all other tested mutant types failed to replicate. Thus, a UG pair at this position is likely required for replication.

The surprising appearance of progeny in systemically infected leaves (indicated in 7:353 grids above the canonical PSTVd secondary structure in Figure 4) in the apparent absence of replication in inoculated leaves can be attributed to reversion of the UU mutation to wild type UG in one infected plant, and reversion of the UA mutation with introduction of new mutations in another infected plant. In these plants, revertants and sequence variants likely arose late in an inoculated leaf, perhaps just prior to harvest. Similar unusual outcomes, where a particular mutant was detected in systemic but not in local inoculated leaves, were observed at six G/U pairs (7:353, 15:347, 35:326, 44:317, 61:299 and 104:254), and in all cases similar explanations apply.

While none of the introduced mutations at 7:353 UG appeared to replicate, we considered the possibility that at least some of the mutants might be replication competent but unable to spread cell-to-cell in the inoculated leaf. As a result, these mutants may not generate sufficient signal for detection by RNA blot. To test this possibility, the four 7:353 mutants were tested in a single cell replication assay using *N. benthamiana* protoplasts. In three independent experiments, no signal indicating replication was detected by blot analysis of RNA from protoplasts inoculated with any of the mutants. By contrast, wild type PSTVd replication (positive control) was readily apparent (Supplementary Figure S4). This indicates that the mutations introduced at UG pair 7:353 blocked replication.

In addition to 7:353 UG, another 14 mutants distributed among eight G/U pairs also did not appear to replicate in inoculated leaves (Figure 4). Six of these mutants were selected for analysis in the *N. benthamiana* protoplast assay: 35:326 UG to UU, CG, and GU; 104:254 UG to CG; and 156:205 GU to GC and UG. Wild type PSTVd and 35:326 UG to UA, which was able to infect inoculated leaves, were included as positive controls. In contrast to results with 7:353 UG mutants, RNA blot analysis suggested that all of the mutants in this group were able to replicate in transfected protoplasts (Supplementary Figure S4). However, progeny sequencing indicated that the 35:326 UG to UA positive control was the only case where the introduced mutation was retained. All other mutant progeny reverted to G/U base pairs. Similar protoplast assay results were obtained in a separate study of five PSTVd loop region mutants that also failed to replicate in inoculated leaves (46). We concluded that mutants that did not produce a detectable signal in inoculated leaves were not replication competent, and that local leaf infection is a valid replication assay. However, for all tested mutants other than those at position 7:353, some limited but undetectable replication must have been possible, after which revertants rapidly dominated the progeny population.

### Analysis of double mutants reveals additional G/U pairs required for PSTVd replication or trafficking

We observed that most G/U pairs next to or near another in the same base-paired stem region were more permissive of mutation (Figure 4), suggesting that the function of one might be compensated by the other. To test this possibility, we separately combined G/U to U/U and G/U to G/C single mutants at adjacent G/U pairs within the same stem to generate double mutants (i.e. U/U + U/U and G/C + G/C). We selected U/U because this base combination is the most flexible of the pairs tested, while G/C is the more rigid of the two canonical WC base pairs. The double mutants were analyzed for replication in local leaves and systemic trafficking as described above.

**Figure 5.**
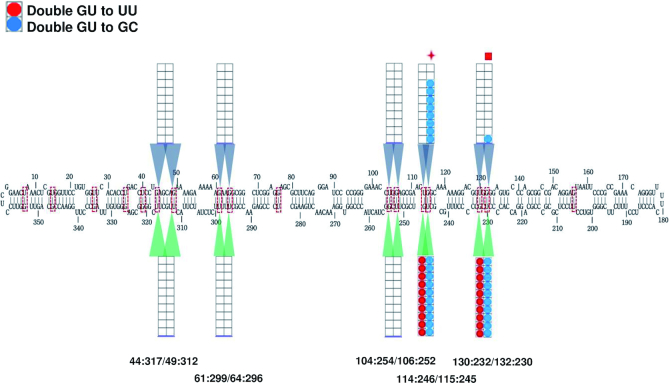
Replication and trafficking rates for PSTVd G/U double mutants. For each stem region containing two G/U pairs, G/U to U/U and G/U to G/C single mutants were separately combined to generate double mutants. The nucleotide coordinates for the coupled G/U pairs are given below the diagram of the canonical PSTVd secondary structure. Ten *N. benthamiana* plants were inoculated with each mutant and RNA from inoculated (local) and systemically infected leaves was evaluated for the presence of PSTVd by RNA blot analysis. For each mutation type, 10 boxes within a grid represent 10 inoculated plants. Lower grids show data for local inoculated leaves, where positive signals indicate replication. Upper grids show data for systemically infected leaves, where positive signals indicate potential systemic trafficking. Colored circles represent infected plants: double G/U to U/U (red); double G/U to G/C (blue). Symbols above the diagram represent results of progeny sequencing from systemically infected leaves. Red cross: retained one introduced mutation, one reverted to G/U. Red square: lost both introduced mutations (reverted to G/U) and acquired a new mutation. For single mutant data, see Figure 4.

As expected, in all cases where one of the single mutants was non-functional, combined mutants also proved non-functional, and mutants that did not replicate in inoculated leaves could not spread to systemic leaves. However, while single G/U to G/C mutants at 44:317 and 49:312 were able to replicate in local inoculated leaves, combining the two mutations abolished local leaf infectivity, suggesting that the presence of at least one G/U pair in the stem bordered by nucleotides 44 and 49 is required for replication (compare grids below the PSTVd structure in Figures 4 and 5). Similarly, single G/U to U/U mutants at 61:299 and 64:296 were replication competent but the double mutant was not, suggesting that at least one G/U pair in the stem spanning nucleotides 60–68 is essential for replication. The same outcome was observed with a 104:254/106:252 G/U to U/U double mutant, located in the stem encompassing nucleotides 103 to 111.

Different results were obtained with combined mutations at adjacent G/U pairs 114:246/115:245 in a short stem spanning nucleotides 114–117, and G/U pairs 130:232/132:230 in the stem between nucleotides 128–134. At these sites, G/U to U/U and G/U to G/C double mutations had no impact on local leaf infectivity, suggesting that the presence of a G/U pair in these stems is not necessary for replication (compare grids below the PSTVd structure in Figures 4 and 5). However, while the single mutants at 114:246/115:245 were capable of systemic spread, both G/U to U/U and G/U to G/C double mutants were unable to traffic to systemic leaves (compare grids above the canonical PSTVd structure in Figures 4 and 5). Although RNA blot analysis suggested an 8/10 systemic infection rate for the 114:246/115:245 G/U to G/C double mutant (Figure 5), sequencing revealed that progeny retained only 115:245 G/C while 114:246 reverted to G/U. These results suggest that the presence of at least one G/U pair in the stem extending from 114–117 is required for systemic trafficking. Due to the inability of 132:230 G/U to U/U and G/U to G/C single mutants to spread systemically without loss of the introduced mutation (Figure 4), 130:232/132:230 double mutants did not provide additional information about systemic spread. In the lone plant that appeared to be systemically infected, progeny had lost both introduced mutations (reverted to G/U) and acquired a new mutation (C259U).

We concluded that the presence of two G/U pairs in the same stem region can obscure their individual importance, providing an explanation for their apparent tolerance to single mutations. Moreover, this analysis yielded evidence suggesting that coupled G/U pairs 44:317/49:312, 61:299/64:296 and 104:254/106:252 are required for replication, while 114:246/115:245 and 130:232/132:230 likely are not. On the other hand, G/U pairs 114:246/115:245 appear to be necessary for systemic trafficking.

### Location and context of G/U pairs is important for function

We observed that when a G/U pair is present alone in a stem region, mutations to G/C resulted in failure to replicate (7:353, 15:347, 27:335, 35:326, 156:205) or in severe impairment of systemic trafficking (76:283) (Figure 4). A possible explanation is that G/C pairs reduce local flexibility, which in turn might inhibit induced conformation recognition between PSTVd RNA and a binding partner. In order to test this possibility, we attempted to restore function. Beginning with the original G/U to G/C mutation that abolished function, new G/U pairs were introduced in the same orientation at other locations within the same stem. Because two G/U pairs in the same stem can functionally compensate, this was not attempted with 61:299, 64:296 or 104:254, where mutations to G/C also abolished replication.

**Figure 6.**
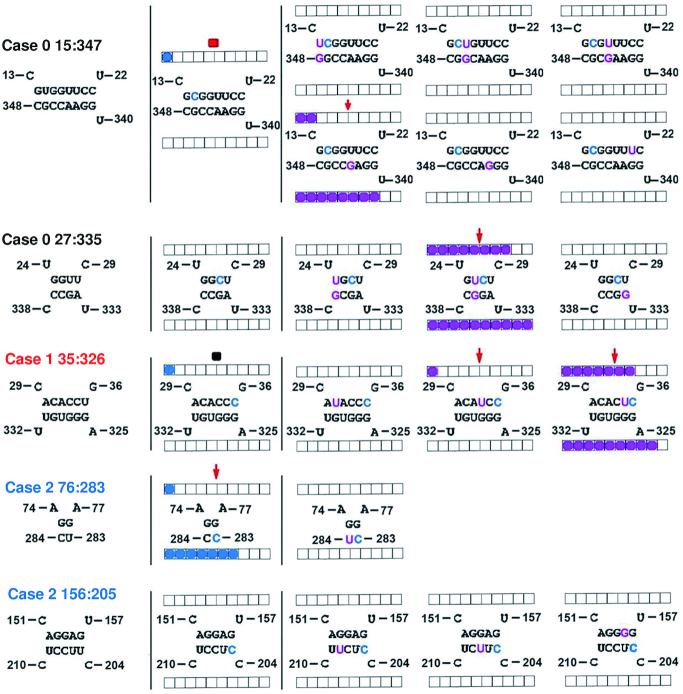
Replication and trafficking rates for PSTVd G/U to G/C mutants with new G/U pairs. Left: secondary structure of wild type PSTVd, with nucleotide coordinates for G/U pairs and sequence context indicated by color. Case 0 (black), Case 1 (red), Case 2 (blue). Middle: G/U to G/C mutant data from Figure 3 showing seriously impaired function. The introduced C nucleotide is highlighted in blue. Right: Bases introduced to generate new G/U pairs are indicated in purple, with the existing G/U to G/C mutation (blue) in the same stem. As in Figures 4 and 5, boxes and solid circles are used to show replication and replication rates for each mutant. Lower horizontal grids show data for local inoculated leaves (replication assay), and upper horizontal grids show data for systemic leaves (potential trafficking). Red arrow: retained introduced mutations without acquiring additional mutations. Red square: did not retain the introduced mutation and acquired new mutations. Black square: reverted to wild type.

Mutation of the UG pair (Case 0) at 15:347 to CG abolished replication. In addition to this original CG mutation, new UG pairs were introduced at 14:348, 16:346, 17:345, 18:344, 19:343, and 20:342. All new pairs were also Case 0 except 14:348, which is in the Case 2 context. Remarkably, the new UG pair at 18:344 restored the ability to replicate and partially restored systemic spread (Figure 6). Similarly, mutation of the UG pair at 27:335 (Case 0) to CG also resulted in loss of replication. In this instance, a new UG pair at 26:336 (Case 0), but not 25:337 (Case 2) or 28:334 (Case 1), nearly completely restored both replication and systemic spread. Mutation of the UG pair at 35:326 (Case 1) destroyed replication, which could not be restored by adding UG pairs at 31:329 or 33:328 (both Case 0). However, replication and trafficking were almost completely recovered by introduction of a UG pair at 34:327 (Case 0). In all cases, progeny obtained from systemically infected leaves retained both the original G/C mutation as well as the new restorative G/U pair, and did not acquire additional mutations.

The UG pair 7:353 (Case 1) differed from other Case 0 or Case 1 G/U pairs tested. Here, restoration of function lost by mutation to a CG pair did not occur when new UG pairs were introduced at the other four sites in the same stem (data not shown). As this region binds Pol II and is essential for replication, it may be that sequence alterations at or near this location are lethal.

Unlike Case 0 and Case 1 G/U pairs, the functionality of two Case 2 G/U to G/C mutants was not restored by addition of new Case 0 or Case 1 G/U pairs. Mutation of the GU pair at 76:283 to GC reduced replication efficiency and severely impaired systemic spread, but addition of a new GU pair (Case 1) at 75:284 had the effect of abolishing replication (Figure 6). Mutation of GU pair 156:205 to GC blocked replication, which could not be restored by new Case 0 GU pairs at 153:208, 154:207 or 155:206.

In summary, with the exception of 7:335 UG which did not tolerate any tested mutation at or near this site, a new G/U pair within one to three bases in the same stem partially or completely restored both replication and trafficking to non-functional G/C mutants at 15:347, 27:335 and 35:326. Gain-of-function provides powerful evidence that a G/U pair in their respective stems is critical for replication and trafficking, and further indicates it is possible to restore function to Case 0 and Case 1 G/U to G/C mutants by introducing new Case 0 G/U pairs. G/U pairs in Case 0 and Case 1 contexts are most likely *cis* WC/WC base pairs. On the other hand, Case 2 G/U pairs 76:283 and 156:205 were not functionally restored by new Case 0 or Case 1 G/U pairs, suggesting that Case 2 conformation imparts a unique property. Possibly at some point in the PSTVd replication cycle these positions require the flexibility to not form a base pair, as Case 2 G/U pairs are known to do from 3D structures.

### G/U pairs 76:283 and 156:205 are required for PSTVd to enter vascular tissue

**Figure 7.**
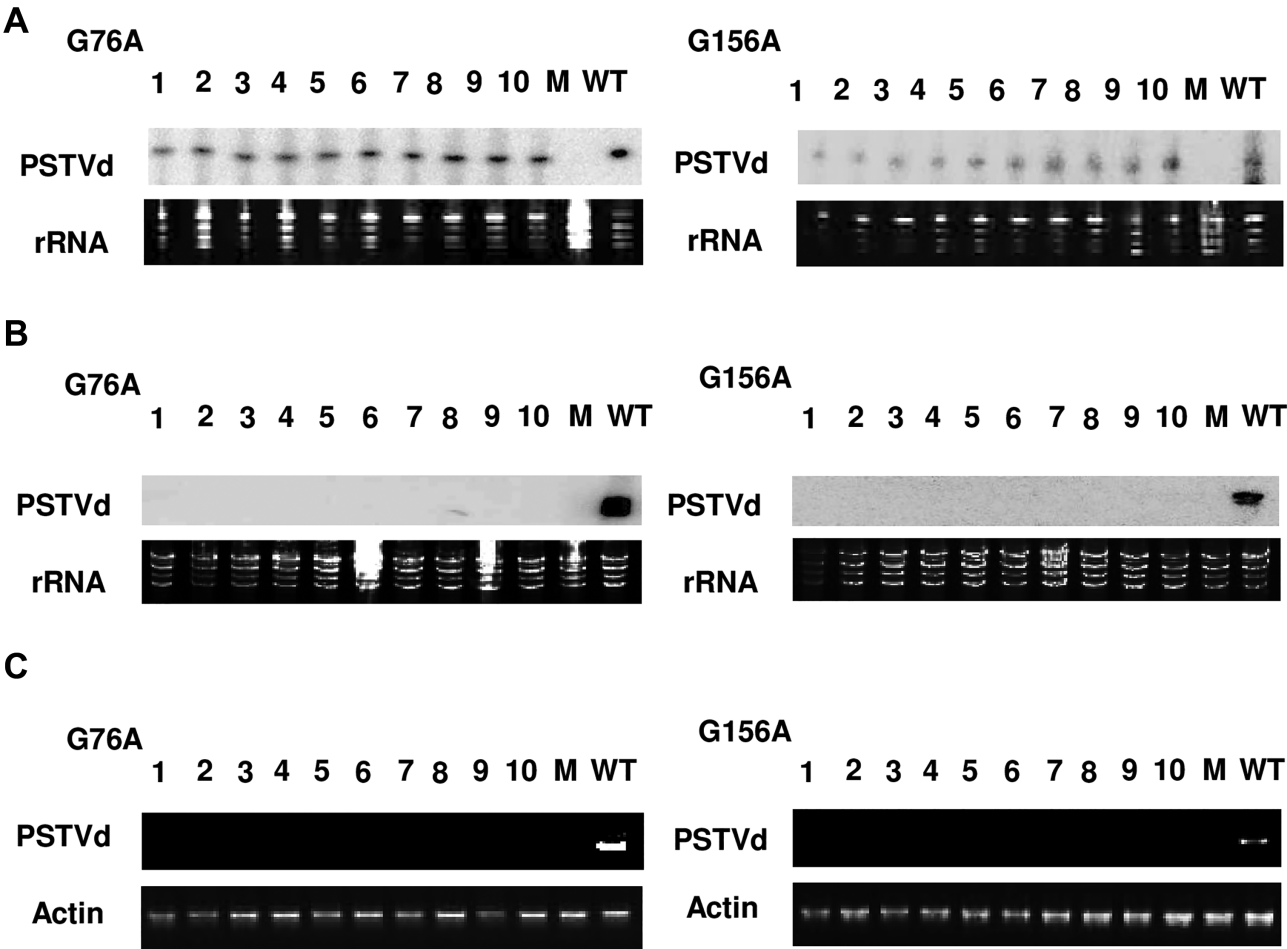
PSTVd G76A and G156A mutants fail to exit rub-inoculated leaves. RNA was collected from (**A**) rub-inoculated leaves, (**B**) systemically infected leaves, or (**C**) petioles of inoculated leaves of 10 plants infected with G76A or G156A. Wild type PSTVd (WT) was a positive control, and mock inoculation (M) was a negative control. (A) RNA blots show a 100% infection rate (10/10 plants) for both mutants in rub-inoculated leaves. (B) Absence of signal on RNA blots indicates both mutants are unable to traffic to upper leaves following rub inoculation. (C) Absence of detectable RT-PCR products indicates that G76A and G156A fail to exit inoculated leaves. Loading controls were ribosomal RNA (A and B) and RT-PCR of actin mRNA (C), detected by ethidium bromide staining. Images are representative of eight (A and B) and three (C) independent experiments.

Because GU to GC mutants at 76:283 and 156:205, which are in the Case 2 context, could not be functionally restored by addition of new GU pairs, we decided to further investigate their biological functions. When these GU pairs were mutated to AU pairs, both mutants (G76A and G156A) were able to replicate in inoculated leaves but were unable to spread to systemic leaves above the inoculation site (Figure 4). We first verified these results in multiple repeat experiments. Again, with each mutant a 100% infection rate (10/10 plants) was invariably observed in inoculated leaves (Figure 7A), but PSTVd RNA was not detected in systemic leaves (Figure 7B). We also performed RT-PCR with PSTVd-specific primers using RNA obtained from the petioles of leaves harvested 10 dpi. While a product of expected size was observed when RNA was obtained from wild type PSTVd-infected plants (positive control), no products were detected from plants inoculated with G76A or G156A (Figure 7C), indicating that the mutants failed to traffic out of inoculated leaves.

Degradation of PSTVd RNA can result in loss of systemic infectivity. To assess their stability, *in vitro* transcripts of G76A and G156A mutants and wild type PSTVd were incubated in buffer or in extracts prepared from uninfected *N. benthamiana* leaves. The amount of RNA remaining over time was determined by RNA blot analysis. We found that while degradation was more rapid in leaf extracts, decay rates for all of the RNAs were similar (Supplementary Figure S5). While this assay does not mimic conditions in cells where PSTVd replicates, it nevertheless demonstrates that the mutant RNAs are reasonably stable. Thus, it is unlikely that reduced stability is responsible for the failure of G76A and G156A to spread to upper leaves.

**Figure 8.**
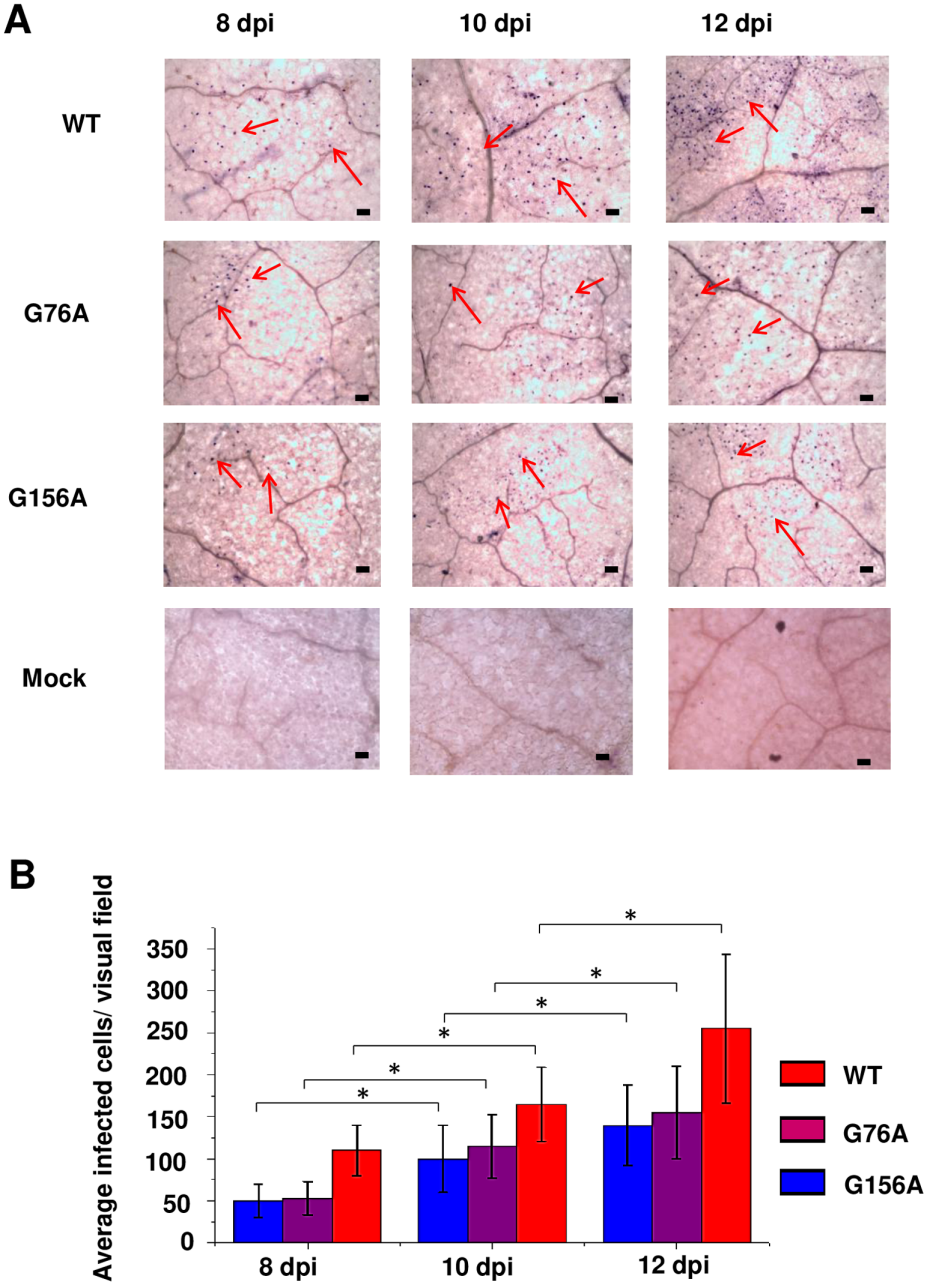
G76A and G156A mutants replicate and spread in rub-inoculated leaves. (**A**) Infection was monitored by whole mount *in situ* hybridization in leaves rub-inoculated with wild type PSTVd (WT), G76A, or G156A at 8, 10 and 12 dpi. Mock inoculation was a negative control. Purple dots (some indicated by red arrows) are viroid hybridization signals in nuclei. Bars = 100 μm. Images for PSTVd WT, G76A, and G156A are representative of >200 visual fields. (**B**). Mean numbers of infected cells per visual field. Asterisks indicate significant differences (*P* < 0.05) as determined by Student's *t* test. Bars indicate standard error of the mean.

Local cell-to-cell spread of viruses and viroids occurs through plasmodesmata that interconnect adjacent cells, and long-distance systemic transport occurs in vascular tissue, typically the phloem. As PSTVd RNA was rub-inoculated onto the upper surfaces of *N. benthamiana* leaves, failure of cell-to-cell transport might prevent a mutant viroid from reaching the vascular tissue, which typically is three to four cell layers beneath the epidermis. To address this question, we first performed whole mount *in situ* hybridization using digoxygenin (DIG)-labeled PSTVd riboprobes with tissue from inoculated leaves harvested 8, 10 and 12 dpi. Whole mount in this case means examining the upper surfaces of small pieces of leaf tissue, rather than thin sections. This method of observation does not permit unambiguous identification of cell types, but most nuclei containing PSTVd signals are likely in epidermal cells. More than 200 visual fields (∼1 mm × 1 mm) randomly selected from the inoculated leaves of 10 plants were examined for each time point. Positive PSTVd hybridization signals were apparent in nuclei of cells infected with wild type PSTVd as well as the G76A and G156A mutants (Figure 8A). For all three, an increase in the number of infected cells per visual field was observed with increased time post-inoculation, indicating successful cell-to-cell spread (Figure 8B). G76A and G156A infected a similar number of cells, which approached or exceeded 50% of the number infected by wild type PSTVd at each time point, an amount more than sufficient to support systemic spread. In a previous study, PSTVd mutants with infection efficiencies as low as 10% of wild type were shown to achieve systemic infection (48).

**Figure 9.**
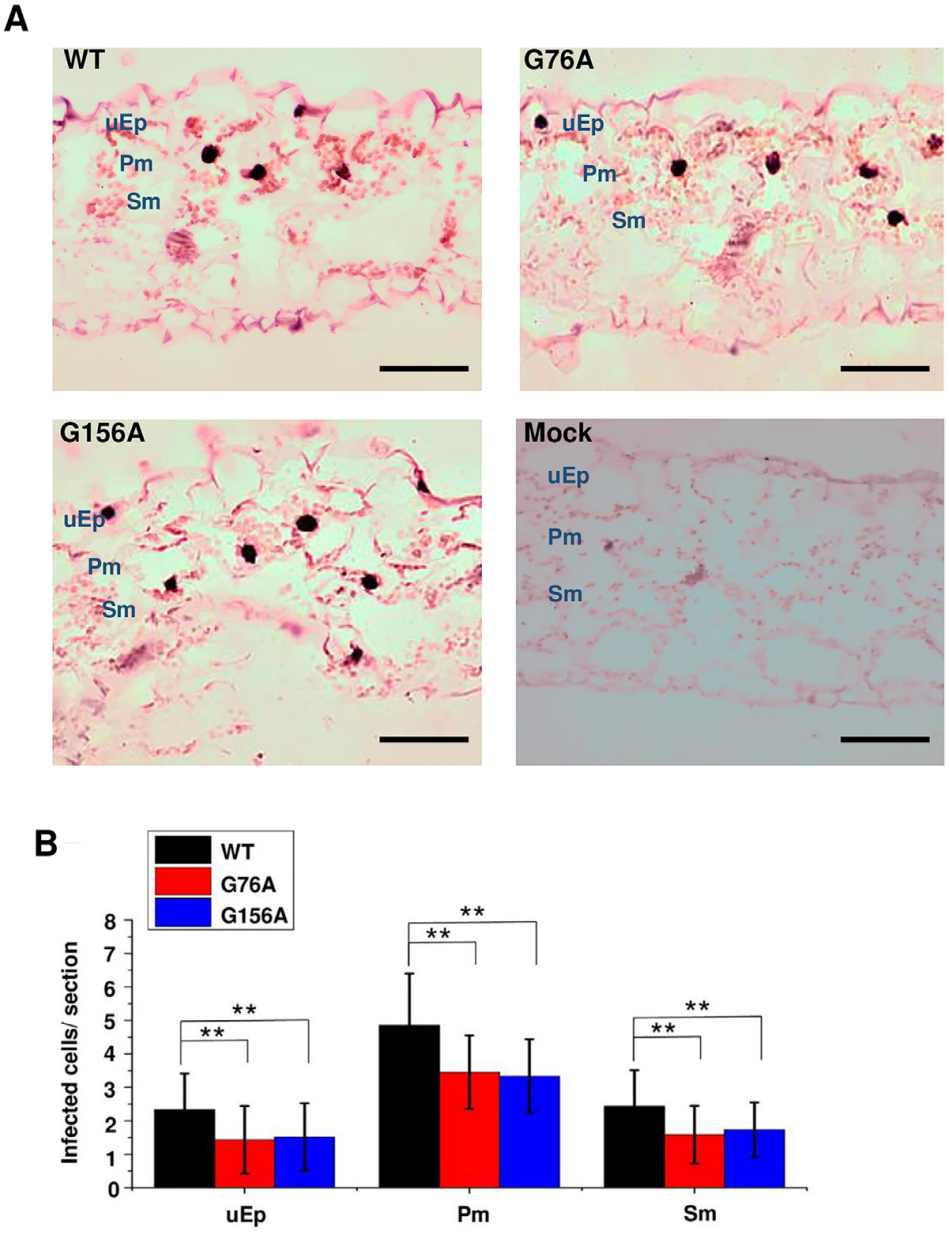
G76A and G156A mutants can spread from epidermal cells into mesophyll cells. (**A**) *In situ* hybridization was performed with transverse sections (12 μm) obtained from mock inoculated leaves (negative control) or leaves rub-inoculated with wild type PSTVd (WT), G76A or G156A. Images are representative of more than 60 sections. Purple dots (red arrows) are viroid hybridization signals in nuclei. uEp, upper epidermis; Pm, palisade mesophyll; Sm, spongy mesophyll. Bars = 100 μm. (**B**) Number of infected cells per leaf section (∼1 × 0.15 mm) in the upper epidermis (uEp), palisade mesophyll (Pm) or spongy mesophyll (Pm) of plants inoculated with WT PSTVd (black), G76A (red) G156A (blue) at 12 dpi. Data were compiled from 40 sections obtained from 20 infected plants. Asterisks indicate significant differences (*P* < 0.01**) as determined by Student's *t* test. Bars indicate standard error of the mean.

Spread through cell types lying between the upper epidermis and vascular tissue is also required for systemic transport. We addressed this question by performing *in situ* hybridization with transverse sections prepared from rub-inoculated leaves. Representative images from 60 sections obtained from 20 plants at 12 dpi are presented in Figure 9. Our rub inoculation method involves abrasive mechanical inoculation of PSTVd *in vitro* transcripts onto the first two true leaves of *N. benthamiana* plants that were previously dusted with carborundum powder. Transverse sectioning of these young inoculated leaves is difficult and most sections do not contain visible vascular tissue, so it was not possible to draw firm conclusions about vascular cell types. However, this analysis clearly showed that wild type PSTVd, G76A and G156A were able to infect cells of the upper epidermis (inoculation site) as well as underlying palisade and spongy mesophyll cells, and so were able to traffic between these cell types. Further, G76A and G156A infected a similar number of cells in each cell type, which in all cases approached or exceeded 50% of the number infected by wild type PSTVd.

**Figure 10.**
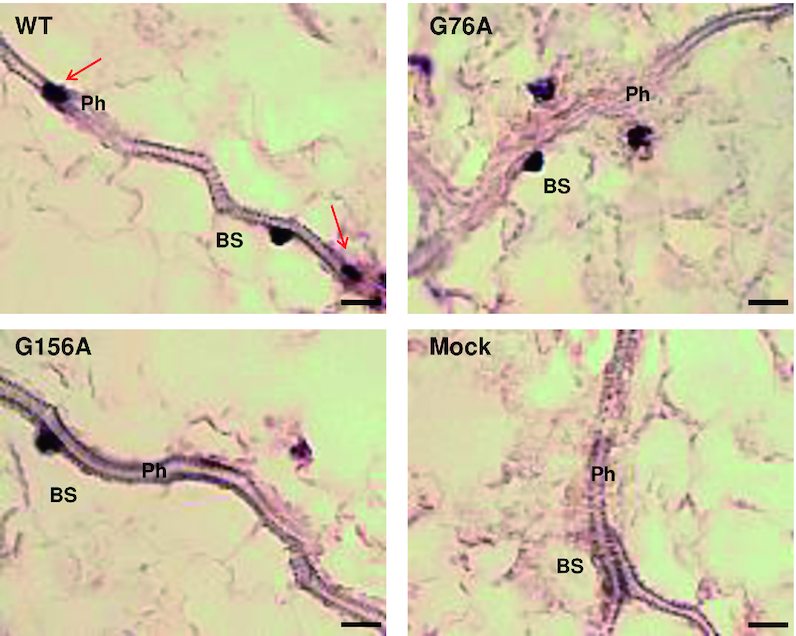
Mutants G76A and G156A fail to traffic into vascular tissue. *In situ* hybridization was carried out with paradermal sections obtained 12 dpi from mock inoculated leaves or leaves rub-inoculated with wild type PSTVd (WT), G76A or G156A. Purple dots are viroid hybridization signals. Note that WT PSTVd accumulates in bundle sheath (BS) and phloem cells (Ph) (red arrows), while the two mutants are found in bundle sheath cells but are absent from the phloem. Images shown are representative of more than 40 sections for each treatment. Bars = 10 μm.

To directly observe vascular tissue, paradermal sections (i.e. parallel to the leaf surface) were prepared for *in situ* hybridization. Approximately 150 sections taken from 60 plants were examined for each treatment. About one-third of these sections contained vascular tissue, and images presented in Figure 10 are representative of 51, 43 and 44 informative sections for wild type PSTVd, G76A, and G156A, respectively. As expected, wild type PSTVd was detected in all cell types, including phloem cells and bundle sheath cells that surround the phloem. By contrast, while PSTVd G76A and G156A were frequently found in bundle sheath cells, they were never observed in phloem cells. We concluded that G76A and G156A are unable to traffic systemically because they likely cannot transit from bundle sheath into phloem cells.

### PSTVd trafficking across the bundle sheath-phloem boundary can be unidirectional

**Figure 11.**
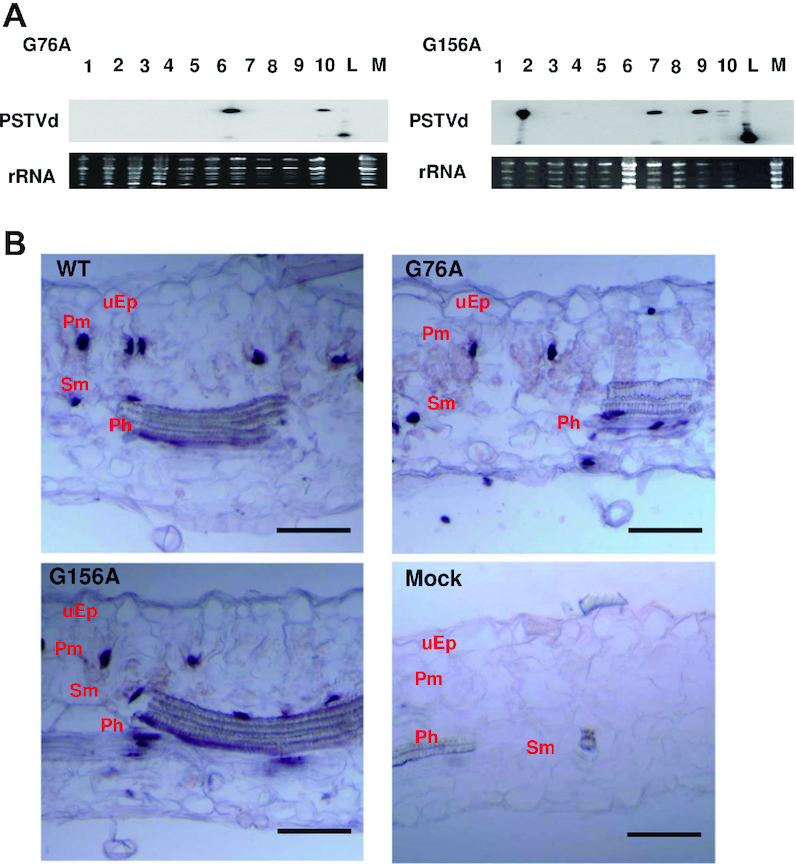
Needle puncture inoculation allows trafficking of G76A and G156A to all cell types in upper leaves. (**A**) RNA was collected from upper leaves of 10 plants 28 days after needle puncture inoculation with G76A and G156A. Linear PSTVd transcript (positive control, L) and RNA from mock inoculated plants (negative control, M) are indicated. Linear PSTVd migrates faster than the circular form that predominates in infected cells. Ribosomal RNA (loading control) was visualized by ethidium bromide staining. RNA blot is from Table 3, experiment 2 (of four). (**B**) *In situ* hybridization was performed with 12 μm transverse sections collected from upper *N. benthamiana* leaves 28 days after needle puncture inoculation with wild type PSTVd (WT), G76A, or G156A. Images for each are representative of >100 sections. Purple dots are viroid hybridization signals. uEp, upper epidermis; Pm, palisade mesophyII; Sm, spongy mesophyII; Ph, phloem. Bars = 100 μm.

PSTVd moves between cells through plasmodesmata which differ between cell types, leading to fine regulation of RNA trafficking. Therefore, we investigated the outcome of PSTVd G76A and G156A infections that bypass the bundle sheath-phloem cell boundary. This was done by delivering circularized G76A and G156A *in vitro* transcripts directly into vascular tissue by needle puncture inoculation of stems and petioles of six week-old plants (46). Leaves above the inoculation sites were harvested after 28 days, and subsequent RNA blot analysis indicated that both mutants were systemically infectious when introduced by this method (Figure 11A). Most of the harvested leaves were formed before inoculation, thus cell types were likely differentiated prior to the arrival of PSTVd. Overall infection rates were low: ∼25% for wild type PSTVd, 12.5% for G76A and 15% for G156A over the course of four experiments. Sequencing full length clones recovered from upper leaf extracts indicated that all G76A and G156A progeny (eight each) retained their original mutations, although for both mutants, half of the progeny also acquired one or more additional mutations at other sites (Table 7). However, as most of the new mutations are unique, they are likely stochastic in nature and unlikely to have compensatory functions. Thus, the G76A and G156A mutants are largely stable.

**Table 7.** Sequences of progeny from plants inoculated with G76A or G156A by needle puncture

	WT	G76A	G76A progeny sequences	G156A	G156A progeny sequences
Experiment 1	2/10	1/10	G76A (2)	0/10	N/A
			G76A/A63U (1)		
Experiment 2	4/10	2/10	G76A (1)	4/10	G156A (1)
			G76A/G68A/C69A (1)		G156A/Δ89A (1)
Experiment 3	1/10	0/10	N/A	2/10	G156A (2)
					G156A/U157C (1)
Experiment 4	3/10	2/10	G76A (1)	1/10	G156A (1)
			G76A/G97U (1)		G156A/C259U (2)
			G76A/C151G (1)		

In each experiment, 10 plants were inoculated by needle puncture with wild type PSTVd (WT), G76A, or G156A. The number of systemically infected plants, determined by blot assay of RNA collected from upper leaves 28 dpi, is indicated. Sixteen mutant progeny were sequenced, and all retained the introduced mutation (bold). However, for each mutant half of the progeny (4/8) also acquired new and mostly unique mutations.

That G76A and G156A are systemically infectious following delivery to vascular tissue by needle puncture implies that they are capable of moving from phloem cells into bundle sheath cells. To confirm this, we examined transverse sections by *in situ* hybridization to monitor the spread of G76A and G156A mutants. Upper systemically infected leaves are easier to section than inoculated leaves, and sections often contain vascular tissue. Remarkably, we found that sections from plants infected with G76A or G156A were virtually identical to those obtained from plants infected with wild type PSTVd. Hybridization signals indicating the presence of G76A and G156A were present in all cell types, including phloem (Figure 11B).

Considered together, our results suggest that PSTVd G76A and G156A mutants are able to traffic from phloem into bundle sheath cells, mesophyll cells, and epidermal cells when delivered to vascular tissue by needle puncture inoculation. However, they cannot transit in the reverse direction, from bundle sheath into phloem cells, when delivered by rub inoculation. We conclude that requirements for RNA trafficking between bundle sheath and phloem are unique and directional, and that G76A and G156A are specifically defective for phloem entry. Although a previous study identified a PSTVd motif necessary for vascular entry (58), to our knowledge this is the first observation of directional trafficking between bundle sheath and phloem cells, and the first instance where defined roles in RNA trafficking have been described for specific G/U pairs.

## DISCUSSION

The role of G/U pairs in the viroid infection cycle has not been extensively explored, and the potential presence of 17 G/U pairs in the canonical PSTVd (intermediate strain) secondary structure offered an opportunity to address this issue as well as how multiple G/U pairs might functionally interact. Although PSTVd secondary structure is well established, a recent whole molecule SHAPE study raised some questions about its true nature and, most relevant to this report, the identity and number of G/U pairs present in the genome (36). Consequently, we began our investigation by performing whole molecule SHAPE with PSTVd-I using the same SHAPE reagent (BzCN) and RNAstructure software. Similar to the previous study, we found that three of the 17 G/U base pairs were not supported. Two of these pairs (49:312 and 114:246), which are in the Case 2 context, exhibited high reactivity to SHAPE reagent (BzCN) (Table 3). Whole molecule SHAPE experiments using NMIA or NAI to probe the highly similar PSTVd-NB variant also did not support G/U base pairs at 49:312 and 114:246 (53). Our analysis of G/U pairs from a very large number of atomic-resolution 3D structures showed that G/U combinations in Case 2 context form cWW wobble base pairs less than 25% of the time, and no base pair >50% of the time (Table 1). Thus, the 49:312 and 114:246 Case 2 combinations may not form wobble pairs, which could explain their high SHAPE reactivity. Nevertheless, functional mutagenesis (discussed below) suggests that these G/U combinations have essential functions in PSTVd replication (49:312) and systemic trafficking (114:246). The remaining unsupported base pair (61:299) displayed low to moderate reactivity and is in Case 0 context, and hence likely forms a wobble base pair. Why it was annotated differently by the structural prediction software is not clear, but again, functional mutagenesis suggests this G/U pair has an essential role in PSTVd infection (see below). Finally, in contrast to whole molecule studies, when SHAPE was performed using nine fragments spanning the PSTVd genome all but one of the 17 G/U pairs in the canonical structure had low SHAPE reactivity, consistent with being base paired. Reactivity of the remaining pair was not determined for experimental reasons. These differential results may be explained by the fact that SHAPE data suggests which nucleotides are involved in cWW base pairs (59), but does not indicate which bases are actually paired with each other. The RNAstructure software then utilizes the SHAPE data to predict the most stable structure, which could vary based on small differences in reactivity between different studies. Another important factor is the flexibility of RNA, which may form alternative structures in solution. Thus, it is possible that SHAPE using genomic fragments will yield somewhat different results than whole molecule SHAPE. In any case, regardless of whether G/U combinations actually form cWW wobble base pairs, our mutagenesis data suggests they are critical for PSTVd infection.

It is also important to consider that the PSTVd genome exists as both positive and negative strands in infected cells, and may fold in different ways to perform essential functions that likely involve interactions with multiple host proteins. In light of this, it is somewhat surprising that results from *in vitro* and *in vivo* whole molecule SHAPE performed with the PSTVd NB variant generated largely similar structures (53), despite the presence of host binding proteins *in vivo* that might shield nucleotides from SHAPE reagents. Possible explanations for the relatively minor differences observed in this study include the asynchronous nature of whole plant infections, where the entire ensemble of infection stages co-exist, and the likely transient nature of viroid-host protein interactions.

G/U and classical WC base pairs have similar structures, but G/U pairs have more conformational flexibility. For functional mutagenesis PSTVd G/U pairs were replaced with A/U and G/C, with A/U being the more flexible of the two. G/U was also replaced by U/U, which has even greater flexibility. In addition, because GU and UG are not self-isosteric, G/U was replaced by U/G. With respect to replication in inoculated leaves, three outcomes were evident (Figure 4). First, at one G/U pair (7:353) known to be located within RNA Pol II and TFIIIA binding sites (19,57), none of the introduced mutations allowed replication, and this result was confirmed in protoplast experiments (Supplementary Figure S4). Second, six pairs (15:347, 27:335, 35:326, 61:299, 64:296 and 156:205) could replicate with substitutions resulting in U/U and/or A/U pairs, but not more rigid G/C pairs or G/U to U/G, suggesting that flexibility at these locations might have a role in replication. Additional studies subsequently confirmed a role in replication for five of these six G/U pairs (see below). Third, at ten pairs (40:321, 44:317, 49:312, 76:283, 104:254, 106:252, 114:246, 115:245, 130:232 and 132:230) three or more mutation types were able to replicate in inoculated leaves. Eight of these more permissive pairs reside in the same WC base-paired stem as another G/U pair, suggesting functional compensation. This also was confirmed by further studies (see below).

Considerably fewer mutants were able to pass the more stringent test of both successful replication and systemic spread. Nevertheless, substitutions resulting in U/U and A/U pairs were again more likely to be functional than G/C mutants. In keeping with non-isostericity, G/U to U/G mutants proved least functional. Only two of these mutants could systemically infect plants, and one of them was severely impaired (Figure 4). In summary, this study identified one G/U pair (7:353) required for replication and six (27:335, 44:317, 61:299, 64:296, 76:283 and 156:205) essential for effective systemic spread. One of these, 61:299, was not annotated as a G/U base pair by whole molecule SHAPE.

Analysis of more permissive sites with two G/U pairs in the same WC base paired stem using double mutants also proved informative. This study confirmed our suspicion that one pair might compensate for the other, and implicated six of the stem co-resident G/U pairs (44:317/49:312, 61:299/64:296, and 104:254/106:252) in PSTVd replication (Figure 5). Three of these G/U pairs (44:317, 61:299, and 64:296) were shown by single mutant analysis to also be essential for systemic spread (see above). Two further co-resident G/U pairs, 114:246/115:245, were found to be critical for systemic spread. It should also be noted that two of the G/U pairs determined to be critical for PSTVd infectivity in this study, 49:312 and 114:246 (both Case 2), were not annotated as G/U base pairs by whole molecule SHAPE.

Gain-of-function experiments carried out on G/U pairs that did not share a stem with another G/U pair were surprisingly successful, and showed that mutations (to G/C) that blocked replication could be at least partially suppressed by addition of a new G/U pair at a different location in the same stem, resulting in both successful replication and systemic spread (Figure 6). This study revealed the critical need for G/U pairs in stems containing 15:347, 27:335 and 35:326. On the other hand, this approach did not restore function to the 7:353 G/U to G/C mutant, likely further highlighting its role in replication. G/C mutations of Case 2 pairs 76:283 and 156:205 also could not be suppressed, suggesting that perhaps Case 2 context imparts some unique property that cannot be mimicked by a Case 0 or Case 1 G/U pair. In this regard, we speculate that Case 0 and Case 1 G/U pairs provide a necessary flexibility that can be achieved by locating a single G/U pair at different positions within a stem, whereas Case 2 G/U pairs have more specific, location-sensitive functions. In some instances, Case 2 G/U function might also involve flexibility to alternatively form a cWW base pair or not to base pair.

To summarize, functional mutagenesis provided evidence that G/U pair 7:353 is required for replication, and that 15:347, 27:335, 35:326, 44:317, 49:312, 61:299, 64:296, 104:254 and 106:252 are also important for this process. Further, the G/U pairs 27:335, 44:317, 61:299, 64:296, 76:283, and 156:205 are critical for systemic spread, and 15:347, 35:326, 114:246 and 115:245 are also important for this activity. Several pairs appear to be involved in both replication and systemic spread, including 15:347, 27:335, 35:326, 44:317, 61:299 and 64:296.

Evidence of important function is lacking for only three sites. G/U pair 40:321, which is present alone in a stem, does not appear to be required for replication and mutation to more rigid G/C does not abolish systemic spread. Single- and double-mutant analysis of stem co-resident 130:232 and 132:230 indicated that these G/U pairs also are not required for replication, and roles in systemic spread could not be established. However, we note that these are the only two G/U pairs where non-isosteric U/G mutants were systemically infectious, suggesting these sites may be less important.

As perhaps might be expected, a survey of 363 PSTVd variants using sequence data available from the National Center for Biotechnology Information showed that nucleotides constituting the 17 G/U pairs are very highly conserved, and in some cases invariant (Supplementary Table S4). For example, no substitutions were observed in G/U pairs shown here to be essential for replication (7:353, Case 1) or entry into the vascular system (76:283 and 156:205, both Case 2). Another Case 2 G/U pair essential for systemic spread, 76:283, also showed no substitutions. On the other hand, base substitutions were also absent from some G/U pairs for which important function has yet to be demonstrated, including 40:321 and 132:230 (both Case 0). Notably, of the six total G/U pairs where no substitutions were observed, three were in Case 2 context. These observations underscore the importance of G/U pairs and the special nature of Case 2 pairs.

Using a novel deep sequencing approach (Figure 3), we also investigated the type and frequency of natural variation at G/U pairs in progeny quasispecies derived from a cloned master sequence. We considered only variants shared by cognate libraries from inoculated (local) and systemically infected leaves, as these were considered more likely to retain both the ability to replicate and traffic systemically. As perhaps might be expected, we found that in variant progeny genomes, which represented only ∼10% of the population, G/U pairs were most often replaced by A/U and G/C pairs (Table 5). However, unlike results of mutagenesis studies where A/U mutants were more likely to be functional, A/U and G/C substitutions were equally likely. A comparison of all nucleotides further revealed that substitutions at classical WC pairs and G/U pairs occurred just over half as often as expected by chance, with substitutions at Case 2 G/U pairs occurring at the lowest rate (Table 6). Thus, substitutions of nucleotides involved in A/U, G/C and G/U pairs is more likely to negatively impact function than replacements in loop regions, which occurred twice as often as expected. We speculate that conservation of G/U pairs is, at least in part, a reflection of their importance for providing conformational flexibility, while canonical WC pairs largely establish overall PSTVd secondary structure by creating relatively rigid stems. As all viroid species have characteristic rod shaped-secondary structures, it seems this feature is critical for replication and systemic spread. In addition, it is probable that specific G/U and canonical WC base pairs mediate important contacts with host factors. Thus, both are crucial for PSTVd viability.

Further comparison of outcomes from deep sequencing PSTVd variants (Table 5) and directed mutagenesis (Figure 4) is informative. Of the 37 total mutations shared in cognate local and systemic libraries, 32 coincide with tested mutations (i.e. G/U to A/U, G/C or U/U). Of these, 20 (62.5%) proved to be replication competent and 11 of these (34.4%) could systemically infect plants. Thus, similar to plant virus populations (54), non-functional variants can be maintained in the presence of wild type PSTVd. However, a considerably larger number of non-functional variants, and variants with non-WC substitutions that were not tested by mutagenesis, were observed in aggregate individual libraries. Thus, our rationale for considering only mutants shared by local and systemic libraries was justified. That said, there are several cases where there is little alignment between deep sequencing and mutagenesis results. To highlight two extreme cases, the UG pair at 7:353 did not tolerate any of the introduced mutations, yet UU and UA variants were observed in progeny populations. This likely reflects support of these variants by wild type PSTVd. At the other extreme, 106:252 was classified as a permissive pair based on mutagenesis results, but no variants meeting our criteria were detected in progeny quasispecies. This may indicate that variants at this position are restricted or simply out-competed by wild type PSTVd.

We also report the first instance of G/U pairs involved in RNA trafficking. Cell-to-cell spread of viroids and viruses occurs through plasmodesmata, which function as channels that provide continuity between plant cells. While the structure, composition, and transit mechanisms of these organelles are incompletely understood, they are known to mediate the transport of small molecules and macromolecules, including proteins, RNAs, and RNA-protein complexes (60–63). Importantly, plasmodesmata are dynamic in nature and can effect changes in permeability that permit transport to be finely regulated. Plasmodesmata also function as gateways to the vascular system, allowing signals to be propagated throughout the plant body but also serving as a highway for systemic spread of viruses and viroids.

Our analysis of G76A and G156A mutants, which impact Case 2 G/U pairs 76:283 and 156:205, revealed that both were unable to transit from bundle sheath cells into phloem cells, although they were competent to move in the opposite direction following inoculation into vascular tissue (Figures 10 and 11). While previous studies showed that a PSTVd loop motif (loop 7) also mediates vascular entry (58), the present study is the first to show that requirements for RNA transit at this boundary are directional and that widely separated sequences and structures are involved. Other work has identified motifs responsible for directional trafficking from bundle sheath to mesophyll cells (37), from palisade to spongy mesophyll (48,64), and from epidermal to palisade mesophyll cells (46). As cell-to-cell movement of PSTVd occurs through plasmodesmata (65), it follows that plasmodesmal gates likely differ between most and perhaps all cell types. It also appears that requirements for passage are often and possibly always directional. The work reported here adds further evidence for directional transit mechanisms, which would allow precise regulation of RNA transport and the establishment of distinct cellular boundaries. In this case, the ability of PSTVd to pass through a plasmodesmal gate between bundle sheath and phloem cells likely involves contacts between a transport and/or plasmodesmal protein(s) at loop 7 (a bulge involving nucleotides 43 and 318) and G/U pairs 76:283 and 156:205. Further study will be required to identify these host factors.

## Supplementary Material

gkaa100_Supplemental_Files
