# Functional analysis reveals G/U pairs critical for replication and trafficking of an infectious non-coding viroid RNA

**DOI:** 10.1093/nar/gkaa1211

**Published:** 2020-12-11

**Authors:** Jian Wu, Cuiji Zhou, James Li, Chun Li, Xiaorong Tao, Neocles B Leontis, Craig L Zirbel, David M Bisaro, Biao Ding

**Affiliations:** Department of Molecular Genetics, Center for Applied Plant Sciences, Center for RNA Biology, and Infectious Diseases Institute, The Ohio State University, Columbus, OH 43210, USA; Graduate Program in Molecular, Cellular, and Developmental Biology, The Ohio State University, Columbus, OH 43210, USA; Department of Molecular Genetics, Center for Applied Plant Sciences, Center for RNA Biology, and Infectious Diseases Institute, The Ohio State University, Columbus, OH 43210, USA; Department of Molecular Genetics, Center for Applied Plant Sciences, Center for RNA Biology, and Infectious Diseases Institute, The Ohio State University, Columbus, OH 43210, USA; Department of Plant Pathology, Nanjing Agricultural University, Nanjing 210095, China; Department of Plant Pathology, Nanjing Agricultural University, Nanjing 210095, China; Department of Chemistry, Bowling Green State University, Bowling Green, OH 43403, USA; Department of Mathematics and Statistics, Bowling Green State University, Bowling Green, OH 43403, USA; Department of Molecular Genetics, Center for Applied Plant Sciences, Center for RNA Biology, and Infectious Diseases Institute, The Ohio State University, Columbus, OH 43210, USA; Graduate Program in Molecular, Cellular, and Developmental Biology, The Ohio State University, Columbus, OH 43210, USA; Department of Molecular Genetics, Center for Applied Plant Sciences, Center for RNA Biology, and Infectious Diseases Institute, The Ohio State University, Columbus, OH 43210, USA; Graduate Program in Molecular, Cellular, and Developmental Biology, The Ohio State University, Columbus, OH 43210, USA

*Nucleic Acids Research*, Volume 48, Issue 6, 06 April 2020, Pages 3134–3155, https://doi.org/10.1093/nar/gkaa100

The Authors wish to report some minor errors in the above article, all of which concern negative controls.

In the original Figure 8A, images for the Mock negative control at 8, 10, and 12 dpi were inadvertently duplicated from Figure 6A of an earlier paper (46). The whole mount experiments in these papers were performed together. The revised figure contains new images for the Mock inoculation control at 8, 10, and 12 dpi.

**Figure 8.**
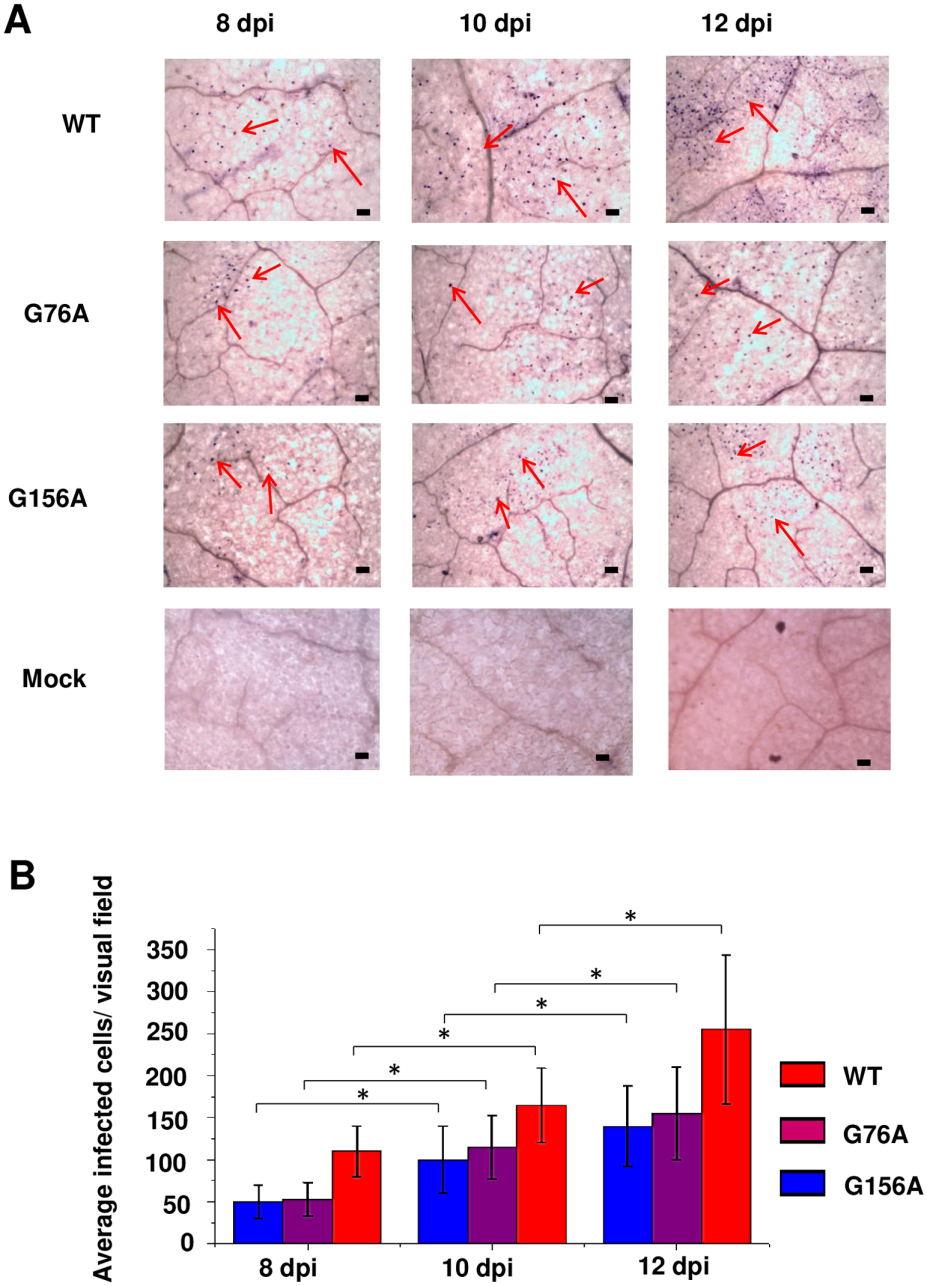
G76A and G156A mutants replicate and spread in rub-inoculated leaves. (**A**) Infection was monitored by whole mount *in situ* hybridization in leaves rub-inoculated with wild type PSTVd (WT), G76A, or G156A at 8, 10 and 12 dpi. Mock inoculation was a negative control. Purple dots (some indicated by red arrows) are viroid hybridization signals in nuclei. Bars = 100 μm. Images for PSTVd WT, G76A, and G156A are representative of >200 visual fields. (**B**). Mean numbers of infected cells per visual field. Asterisks indicate significant differences (*P* < 0.05) as determined by Student's *t* test. Bars indicate standard error of the mean.

In the original Figure 9A, the Mock negative control was inadvertently duplicated from Figure 7 of an earlier paper (46). The transverse section experiments in these papers were performed together. The revised figure contains a new Mock control image.

**Figure 9.**
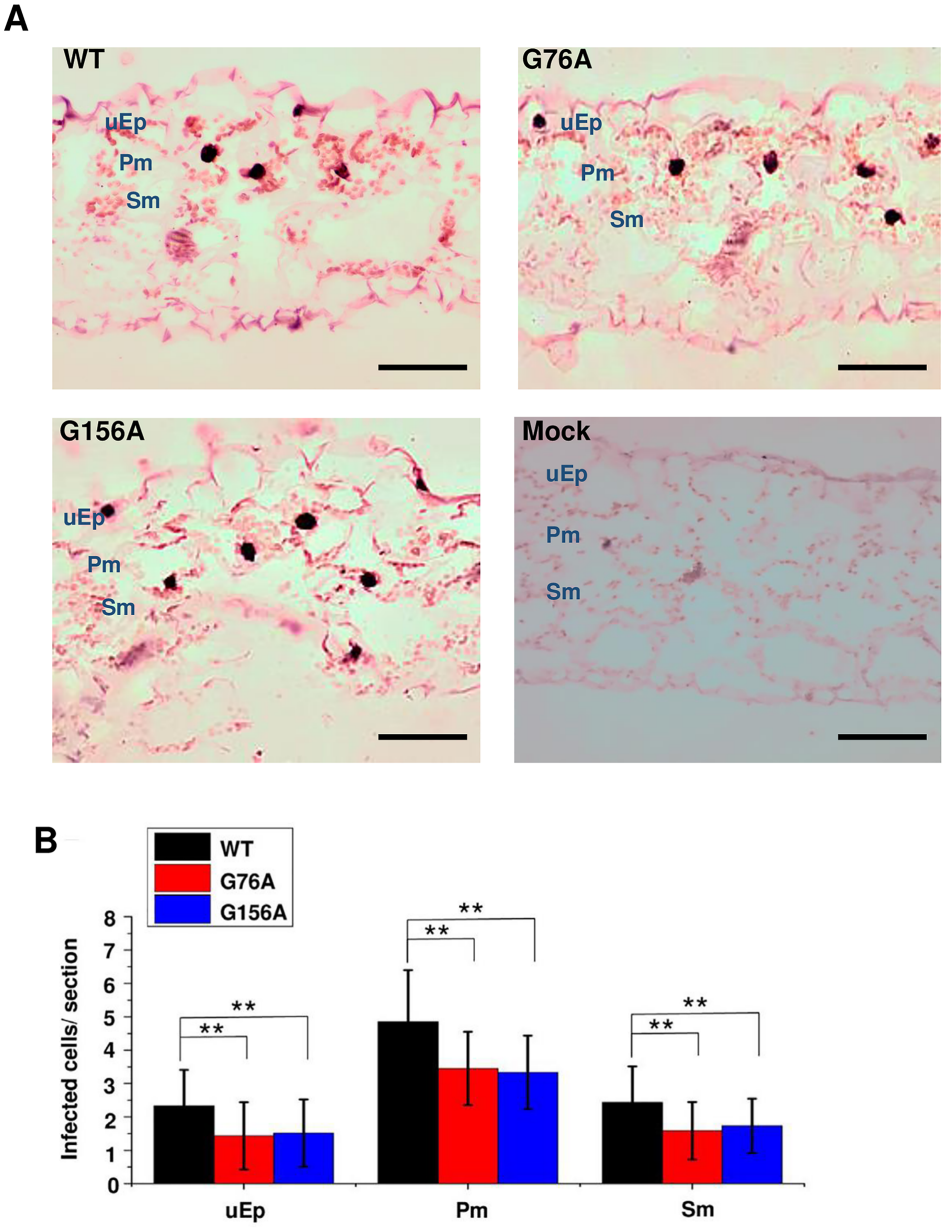
G76A and G156A mutants can spread from epidermal cells into mesophyll cells. (**A**) *In situ* hybridization was performed with transverse sections (12 μm) obtained from mock inoculated leaves (negative control) or leaves rub-inoculated with wild type PSTVd (WT), G76A or G156A. Images are representative of more than 60 sections. Purple dots (red arrows) are viroid hybridization signals in nuclei. uEp, upper epidermis; Pm, palisade mesophyll; Sm, spongy mesophyll. Bars = 100 μm. (**B**) Number of infected cells per leaf section (∼1 × 0.15 mm) in the upper epidermis (uEp), palisade mesophyll (Pm) or spongy mesophyll (Pm) of plants inoculated with WT PSTVd (black), G76A (red) G156A (blue) at 12 dpi. Data were compiled from 40 sections obtained from 20 infected plants. Asterisks indicate significant differences (*P* < 0.01**) as determined by Student's *t* test. Bars indicate standard error of the mean.

The published article has been updated. As Mock inoculation is a negative control, images are devoid of hybridization signals, and these errors have no impact on the conclusions reached in the article.
