# Supplementary material for: Functional analysis reveals G/U pairs critical for replication and trafficking of an infectious non-coding viroid RNA
Source: Nucleic Acids Res. 2020 Feb 21;48(6):3134–55. doi: 10.1093/nar/gkaa100 (PMC7102988; doi:10.1093/nar/gkaa100)
Supplement: gkaa100_Supplemental_Files [file gkaa100_supplemental_files.zip › GU-supplemental Figures.pdf]

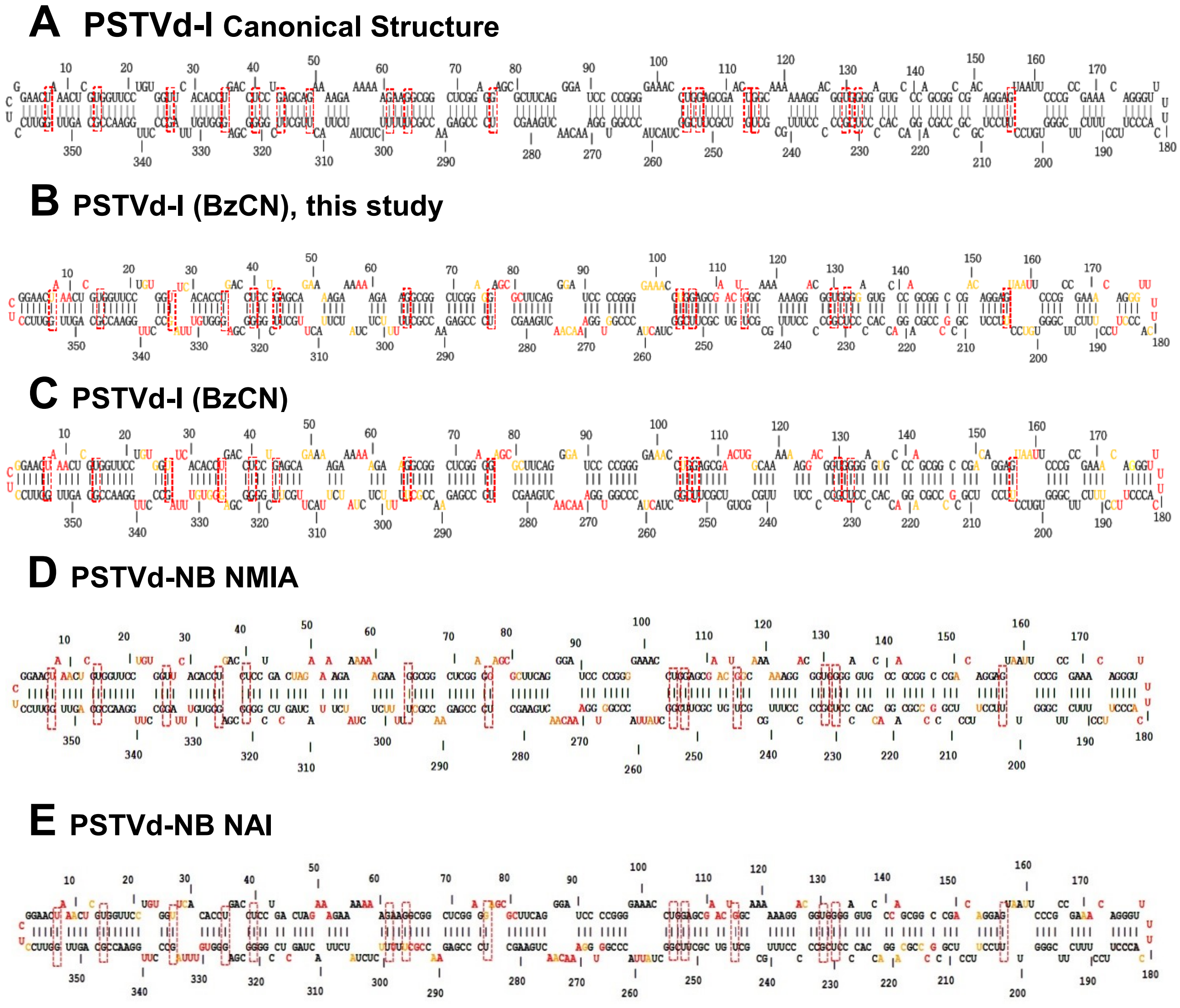


**Supplementary Figure S1.** Comparison of four PSTVd secondary structures. (**A**) The canonical secondary structure of PSTVd-I (intermediate strain) contains 17 G/U base pairs. (**B**) The secondary structure of PSTVd-I from this study, obtained using whole molecule SHAPE with BzCN, predicts 14 G/U pairs, lacking those at positions 49:312, 61:299, and 114:246 in the canonical structure. (**C**) The whole molecule PSTVd-I SHAPE structure of Adkar-Purushothama *et al*. (2015) obtained using BzCN contains 13 G/U pairs, lacking one at position 115:245 in our structure (B). (**D**). The whole molecule PSTVd-NB variant SHAPE structure of López-Carrasco and Flores (2017) obtained using NMIA. The NB variant does not have a G/U pair found at position 44:317 in PSTVd-I due to a mutation that results in a G/C pair. Otherwise this structure predicts 13 G/U pairs, missing the same three as our structure (49:312, 61:299, and 114:246). **(E)** The whole molecule PSTVd-NB variant SHAPE structure of López-Carrasco and Flores (2017) obtained using NAI contains 14 G/U pairs, lacking those at positions 49:312 and 114:246 in the canonical PSTVd-I structure. G/U pairs are identified by red boxes. SHAPE reactivity is indicated by nucleotide color: red = high reactivity (>0.85), orange = intermediate (0.40-0.85), black = low (0-0.40).

Adkar-Purushothama, C.R., Brosseau, C., Giguère, T., Sano, T., Moffett, P. and Perreault, J.-P. (2015) Small RNA derived from the virulence modulating region of *Potato spindle tuber viroid* silences *callose synthase* genes of tomato plants. *Plant Cell* **27**, 2178-2194.

López-Carrasco, A. and Flores, R. (2017) Dissecting the secondary structure of the circular RNA of a viroid *in vivo*: A "naked" rod-like conformation similar but not identical to that observed *in vitro*. *RNA Biology* **14**, 1046-1054.

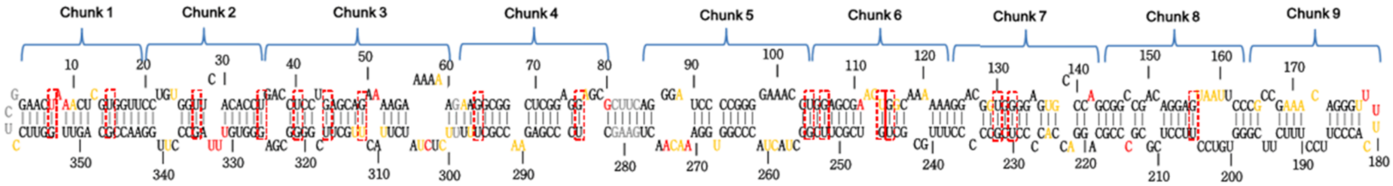


**Supplementary Figure S2.** PSTVd secondary structure obtained by SHAPE using partial genome fragments. In collaboration with the EteRNA project (Lee *et al.*, 2013), the PSTVd genome was divided into 9 fragments (Chunks). SHAPE was performed using NMIA, with five replicates for each fragment. Three different colors are used to represent the averaged SHAPE reactivities, which are superimposed on the canonical structure. Red = high reactivity; orange = intermediate; black = low. Gray = data not available. This analysis predicted 16 of the 17 G/U pairs in the canonical structure. The 16 G/U pairs are indicated by red boxes. Reactivities of nucleotides at the remaining position (61:299) could not be determined due to their location at the junction between Chunks 3 and 4.

Lee, J., Kladwang, W., Lee, M., Cantu, D., Azizyan, M., Kim, H., Limpaecher, A., Gaikwad, S., Yoon, S., Treuille, T., Das, R., and EteRNA Participants (2014) RNA design rules from a massive open laboratory. *Proc. Natl. Acad. Sci. USA*, **111**, 2122-2127.

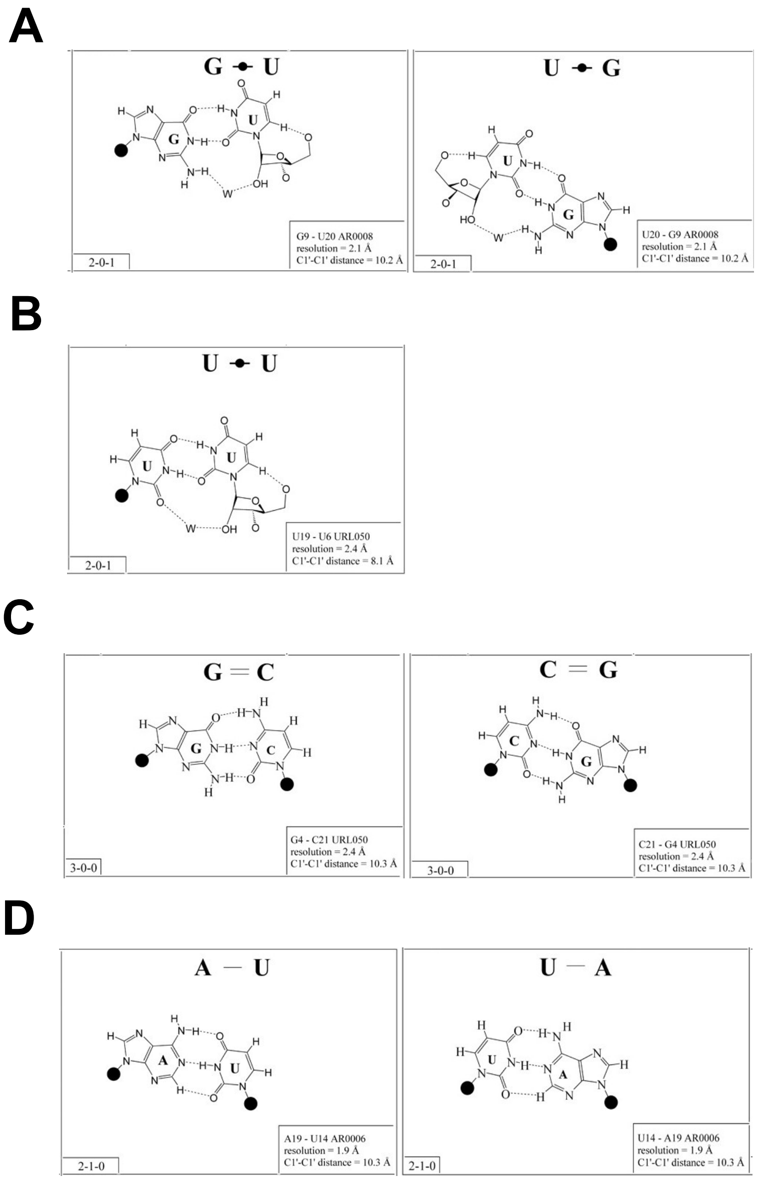


**Supplementary Figure S3**. Schematic diagram of cWW G/U **(A)**, UU **(B)**, G/C **(C)** and A/U **(D)** base pairs. Numbers in the lower left hand corner of each panel describe hydrogen bonding. The first is the number of hydrogen bonds between two nitrogen- or oxygen-containing groups, the second is the number of hydrogen bonds involving polarized C-H groups, and the third is the number of bridging water molecules (W). Numbers in the lower right corner provide information about the X-ray structures used to generate these illustrations.

Images were modified from: Leontis, N.B., Stombaugh, J., and Westhof, E. (2002) The non-Watson-Crick base pairs and their associated isostericity matrices. *Nucleic Acids Research* 30: 3497-3531.

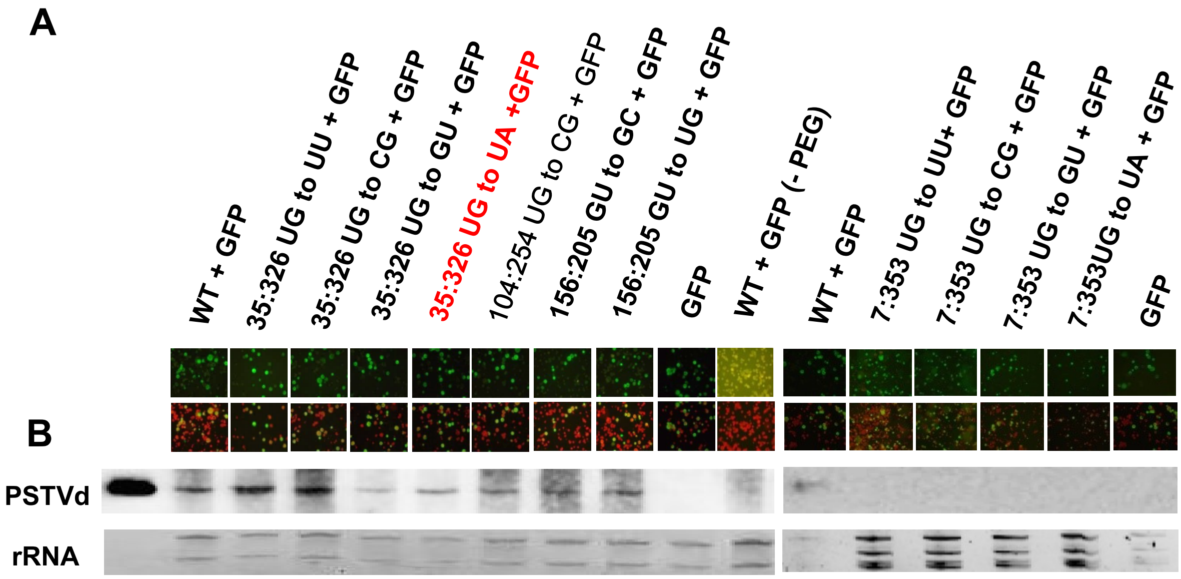


**Supplementary Figure S4.** Replication of G/U mutants in protoplasts. Ten mutants that did not replicate in inoculated leaves were transfected into *N. benthamiana* protoplasts. Inocula consisted of 6 µg of (+)- PSTVd transcripts and 20 µg of a plasmid encoding GFP, which served as an indicator of polyethylene glycol (PEG)-mediated transfection efficiency. Wild type (WT) PSTVd and 35:326 UG to UA (highlighted in red), which showed a 100% infection rate in inoculated leaves, were positive controls. GFP plasmid alone, and WT plus GFP plasmid without PEG, were negative controls. (**A**) Protoplasts were photographed ~18 hours post-transfection in a fluorescence microscope using a filter to block red chlorophyll autofluorescence and image GFP-expressing cells (top panels). Green, blue, and red channels were used to observe all cells (bottom panels). Transfection efficiencies were similar in all cases (~30 to 40%). (**B**) PSTVd RNAs were detected by RNA blot analysis. A circularized PSTVd transcript was used to show the position of circular form PSTVd (first lane, left). Ribosomal RNA (rRNA), visualized by ethidium bromide staining, was a loading control. All mutants at G/U pair 7:353 failed to replicate. All other mutants appeared to replicate, but progeny sequencing (one clone each) indicated that all mutants except positive control 35:326 UG to UA (red) had reverted to UG pairs. Images are representative of three experiments.

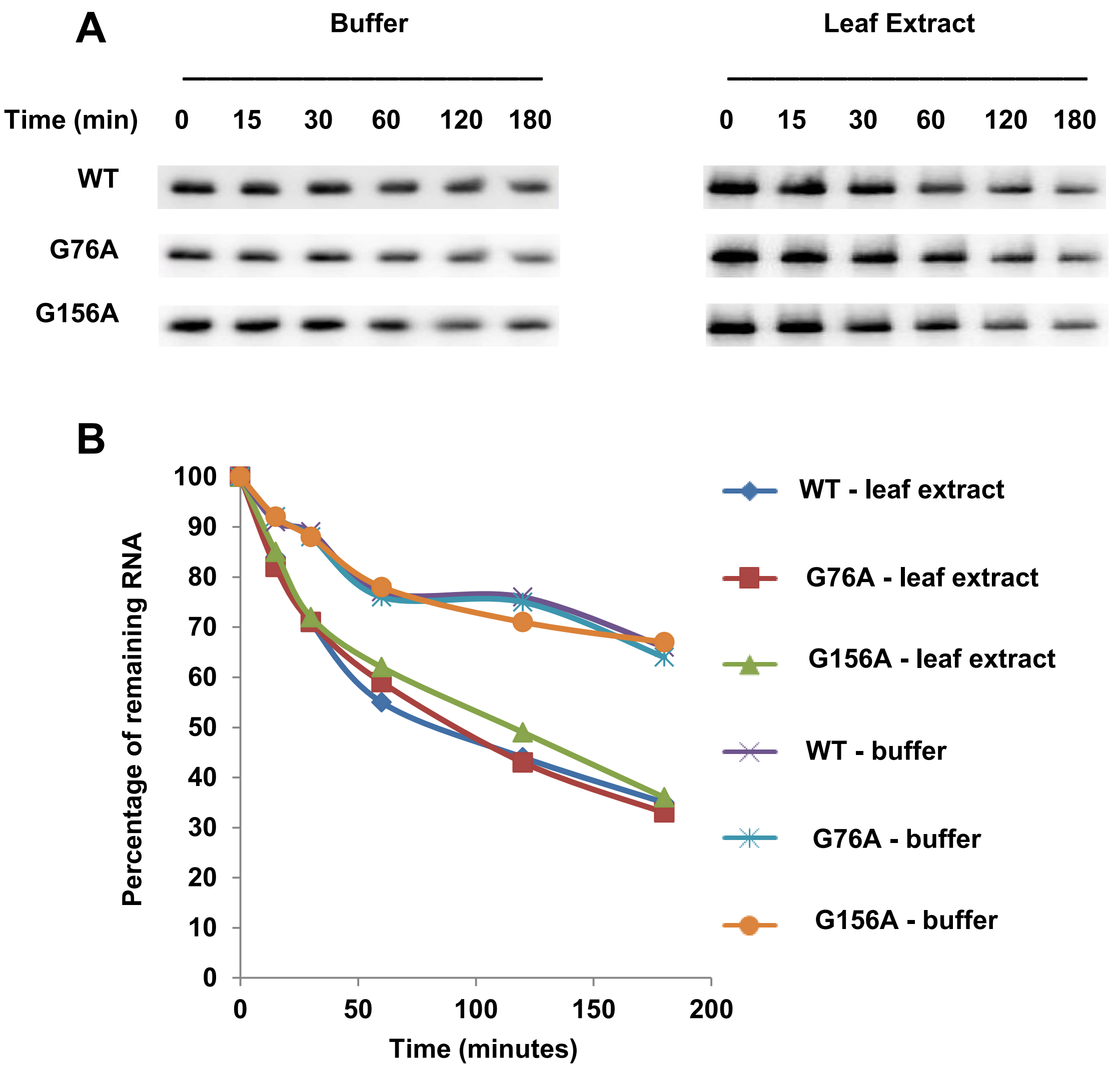


**Figure S5.** G76A, G156A, and wild type PSTVd RNAs have similar stabilities. (**A**) RNA blots of degradation assays performed at 28° C in buffer (20 mM Tris-HCl, pH=7.5, 150 mM NaCl, 10 mM phenylmethylsulfonyl fluoride) or uninfected *N. benthamiana* leaf extract prepared with the same buffer. (**B**) Percentage of remaining wild type and mutant RNAs over time was determined using Quantity One software. Data are representative of three independent experiments.
