# Supplementary material for: Functional analysis reveals G/U pairs critical for replication and trafficking of an infectious non-coding viroid RNA
Source: Nucleic Acids Res. 2020 Feb 21;48(6):3134–55. doi: 10.1093/nar/gkaa100 (PMC7102988; doi:10.1093/nar/gkaa100)
Supplement: gkaa100_Supplemental_Files [file gkaa100_supplemental_files.zip › GU-supplemental tables.pdf]

Supplementary Table S1. Normalized reactivities for PSTVd nucleotides determined by whole molecule SHAPE

Positions	Nucleotides	SHAPE reactivity	Positions	Nucleotides	SHAPE reactivity
1	C	3.27	181	C	0.87
2	G	0.27	182	A	0.07
3	G	0.01	183	C	0.16
4	A	0.00	184	C	0.06
5	A	0.00	185	C	0.46
6	C	0.12	186	U	1.33
7	U	0.77	187	U	0.97
8	A	9.70	188	C	0.23
9	A	2.19	189	C	0.23
10	A	0.95	190	U	0.60
11	C	0.28	191	U	0.23
12	U	0.32	192	U	0.16
13	C	0.56	193	C	0.30
14	G	0.09	194	U	0.35
15	U	0.07	195	U	0.20
16	G	0.04	196	C	0.16
17	G	0.04	197	G	0.02
18	U	0.04	198	G	0.11
19	U	0.03	199	G	0.19
20	C	0.01	200	U	0.11
21	C	0.15	201	G	0.51
22	U	0.04	202	U	0.34
23	G	0.49	203	C	0.15
24	U	7.99	204	C	0.08
25	G	0.42	205	U	0.76
26	G	0.27	206	U	0.08
27	U	0.77	207	C	0.31
28	U	0.95	208	C	0.08
29	C	0.90	209	U	0.22
30	A	0.01	210	C	0.15
31	C	0.03	211	G	0.02
32	A	0.01	212	C	0.00
33	C	0.01	213	G	2.56
34	C	0.09	214	C	1.07
35	U	0.12	215	C	0.27
36	G	0.61	216	C	0.02
37	A	0.19	217	G	0.00
38	C	0.03	218	C	0.14
39	C	0.28	219	A	0.13
40	U	0.07	220	G	0.00
41	C	0.01	221	G	0.27
42	C	0.10	222	A	1.36
43	U	0.67	223	C	0.00
44	G	0.15	224	C	0.17
45	A	0.04	225	A	0.03
46	G	0.02	226	C	0.04
47	C	0.00	227	C	0.00
48	A	0.29	228	C	0.03

49	G	0.57	229	C	0.11
50	A	0.50	230	U	0.25
51	A	0.31	231	C	0.08
52	A	0.49	232	G	0.02
53	A	0.15	233	C	0.01
54	G	0.05	234	C	0.12
55	A	0.01	235	C	0.07
56	A	0.00	236	C	0.04
57	A	0.37	237	C	0.00
58	A	0.88	238	U	0.09
59	A	0.97	239	U	0.25
60	A	0.01	240	U	0.24
61	G	0.05	241	G	0.08
62	A	0.10	242	C	0.09
63	A	0.06	243	G	0.04
64	G	0.03	244	C	0.12
65	G	0.05	245	U	0.01
66	C	0.01	246	G	0.07
67	G	0.08	247	U	0.01
68	G	0.08	248	C	0.13
69	C	0.12	249	G	0.20
70	U	0.09	250	C	0.09
71	C	0.02	251	U	0.12
72	G	0.03	252	U	0.10
73	G	0.17	253	C	0.14
74	A	0.87	254	G	0.17
75	G	0.27	255	G	0.25
76	G	0.20	256	C	0.03
77	A	0.66	257	U	0.05
78	G	2.08	258	A	0.29
79	C	4.34	259	C	3.17
80	G	4.04	260	U	0.64
81	C	0.98	261	A	0.15
82	U	0.39	262	C	0.09
83	U	0.07	263	C	0.00
84	C	0.03	264	C	0.00
85	A	0.00	265	G	0.05
86	G	0.20	266	G	0.46
87	G	0.28	267	U	6.02
88	G	0.51	268	G	0.15
89	A	0.52	269	G	0.09
90	U	0.01	270	A	1.24
91	C	0.02	271	A	14.18
92	C	0.01	272	A	0.82
93	C	0.02	273	C	0.88
94	C	0.00	274	A	0.84
95	G	0.09	275	A	1.26
96	G	0.08	276	C	0.04
97	G	0.12	277	U	0.01
98	G	0.27	278	G	0.00
99	A	0.53	279	A	0.02
100	A	0.65	280	A	0.12
101	A	0.46	281	G	0.03
102	C	0.09	282	C	0.04

103	C	0.34	283	U	0.01
104	U	0.56	284	C	0.00
105	G	0.19	285	C	0.03
106	G	0.12	286	C	0.03
107	A	0.25	287	G	0.03
108	G	0.02	288	A	0.02
109	C	0.00	289	G	0.05
110	G	1.45	290	A	0.21
111	A	2.30	291	A	0.35
112	A	1.51	292	C	0.01
113	C	1.38	293	C	0.03
114	U	6.82	294	G	0.05
115	G	1.06	295	C	0.18
116	G	0.25	296	U	0.10
117	C	0.00	297	U	0.02
118	A	0.00	298	U	1.29
119	A	0.06	299	U	0.68
120	A	0.06	300	U	0.42
121	A	0.10	301	C	0.03
122	A	0.14	302	U	0.01
123	A	0.02	303	C	0.08
124	G	0.02	304	U	0.41
125	G	1.41	305	A	0.46
126	A	2.90	306	U	0.00
127	C	1.00	307	C	0.02
128	G	0.04	308	U	0.27
129	G	0.26	309	U	0.86
130	U	0.14	310	A	0.26
131	G	0.12	311	C	0.16
132	G	0.00	312	U	2.36
133	G	0.13	313	U	4.31
134	G	0.46	314	G	0.13
135	A	0.24	315	C	0.04
136	G	0.11	316	U	0.15
137	U	0.08	317	U	0.18
138	G	0.08	318	C	0.16
139	C	0.23	319	G	0.01
140	C	0.09	320	G	0.01
141	C	0.29	321	G	0.29
142	A	1.94	322	G	0.29
143	G	0.24	323	C	0.24
144	C	0.05	324	G	0.34
145	G	0.01	325	A	1.06
146	G	0.22	326	G	0.85
147	C	0.03	327	G	0.03
148	C	0.07	328	G	0.73
149	G	0.08	329	U	0.63
150	A	0.73	330	G	1.06
151	C	0.65	331	U	2.96
152	A	0.04	332	U	1.56
153	G	0.05	333	U	4.36
154	G	0.10	334	A	0.49
155	A	0.02	335	G	0.38
156	G	0.13	336	C	0.00

157	U	0.51	337	C	0.04
158	A	0.73	338	C	0.31
159	A	0.65	339	U	2.38
160	U	0.99	340	U	3.23
161	U	0.16	341	G	0.20
162	C	0.01	342	G	0.04
163	C	0.00	343	A	0.06
164	C	0.00	344	A	0.08
165	G	0.10	345	C	0.06
166	C	0.10	346	C	0.03
167	C	0.06	347	G	0.13
168	G	0.10	348	C	0.04
169	A	0.07	349	A	0.04
170	A	0.06	350	G	0.06
171	A	0.49	351	U	0.08
172	C	1.11	352	U	0.14
173	A	0.05	353	G	0.05
174	G	0.08	354	G	0.03
175	G	0.80	355	U	0.01
176	G	0.48	356	U	0.01
177	U	4.05	357	C	0.36
178	U	4.27	358	C	1.30
179	U	1.05	359	U	2.89
180	U	1.92			

Whole molecule SHAPE was performed in triplicate on two full-length PSTVd RNA strands beginning at nucleotides 175 and 321. Averaged values are shown. G/U nucleotides highlighted in yellow were judged to be base paired by this analysis. G/U nucleotides highlighted in green are base paired in the canonical PSTVd structure but judged not to be base paired by this analysis. SHAPE reactivities: High = >0.85, Intermediate = 0.40-0.85, Low = 0-0.40.

Supplementary Table S2. Normalized SHAPE reactivities for PSTVd nucleotides from partial sequences

Positions	Nucleotides	Reactivities	Positions	Nucleotides	Reactivities
1	C	ND	181	C	0.76
2	G	ND	182	A	0.27
3	G	0.05	183	C	0.17
4	A	0.00	184	C	0.08
5	A	0.03	185	C	0.05
6	C	0.10	186	U	0.09
7	U	0.99	187	U	0.23
8	A	1.62	188	C	0.26
9	A	0.87	189	C	0.13
10	A	0.48	190	U	0.16
11	C	0.23	191	U	0.24
12	U	0.25	192	U	0.07
13	C	0.75	193	C	0.12
14	G	0.10	194	U	0.18
15	U	0.08	195	U	0.16
16	G	0.04	196	C	0.07
17	G	0.08	197	G	0.16
18	U	0.08	198	G	0.20
19	U	0.00	199	G	0.03
20	C	0.12	200	U	0.10
21	C	0.17	201	G	0.23
22	U	0.15	202	U	0.20
23	G	0.25	203	C	0.09
24	U	0.48	204	C	0.02
25	G	0.07	205	U	0.32
26	G	0.05	206	U	0.12
27	U	0.12	207	C	0.05
28	U	0.24	208	C	0.03
29	C	0.31	209	U	0.03
30	A	0.06	210	C	0.10
31	C	0.07	211	G	0.11
32	A	0.04	212	C	0.29
33	C	0.04	213	G	0.18
34	C	0.01	214	C	0.90
35	U	0.05	215	C	0.17
36	G	0.21	216	C	0.13
37	A	0.14	217	G	0.06
38	C	0.03	218	C	0.19
39	C	0.05	219	A	0.10
40	U	0.10	220	G	0.10
41	C	0.04	221	G	0.19
42	C	0.09	222	A	0.40
43	U	0.13	223	C	0.24
44	G	0.05	224	C	0.26
45	A	0.01	225	A	0.43
46	G	0.03	226	C	0.15
47	C	0.05	227	C	0.02
48	A	0.20	228	C	0.01
49	G	0.22	229	C	0.07

50	A	0.82	230	U	0.03
51	A	1.02	231	C	0.03
52	A	0.31	232	G	0.02
53	A	0.15	233	C	0.02
54	G	0.03	234	C	0.04
55	A	0.10	235	C	0.09
56	A	0.10	236	C	0.03
57	A	0.30	237	C	0.15
58	A	0.29	238	U	0.21
59	A	0.46	239	U	0.30
60	A	0.29	240	U	0.29
61	G	ND	241	G	0.18
62	A	0.44	242	C	0.18
63	A	0.30	243	G	0.05
64	G	0.24	244	C	0.07
65	G	0.07	245	U	0.06
66	C	0.02	246	G	0.22
67	G	0.02	247	U	0.16
68	G	0.02	248	C	0.06
69	C	0.00	249	G	0.02
70	U	0.01	250	C	0.03
71	C	0.01	251	U	0.07
72	G	0.06	252	U	0.00
73	G	0.13	253	C	0.18
74	A	0.16	254	G	0.06
75	G	0.31	255	G	0.03
76	G	0.35	256	C	0.17
77	A	0.42	257	U	0.58
78	G	0.06	258	A	0.19
79	C	0.35	259	C	0.58
80	G	2.00	260	U	0.44
81	C	ND	261	A	0.13
82	U	ND	262	C	0.00
83	U	ND	263	C	0.03
84	C	ND	264	C	0.00
85	A	0.30	265	G	0.01
86	G	0.19	266	G	0.04
87	G	0.18	267	U	0.56
88	G	0.33	268	G	0.11
89	A	0.50	269	G	0.14
90	U	0.06	270	A	0.13
91	C	0.03	271	A	2.29
92	C	0.00	272	A	0.53
93	C	0.02	273	C	0.59
94	C	0.00	274	A	0.85
95	G	0.02	275	A	0.38
96	G	0.00	276	C	0.29
97	G	0.02	277	U	0.28
98	G	0.05	278	G	ND
99	A	0.28	279	A	ND
100	A	0.18	280	A	ND
101	A	0.14	281	G	ND
102	C	0.01	282	C	0.05
103	C	0.00	283	U	0.05

104	U	0.20	284	C	0.02
105	G	0.14	285	C	0.02
106	G	0.10	286	C	0.03
107	A	0.16	287	G	0.14
108	G	0.03	288	A	0.06
109	C	0.03	289	G	0.13
110	G	0.20	290	A	0.78
111	A	0.87	291	A	0.71
112	A	0.56	292	C	0.23
113	C	0.42	293	C	0.05
114	U	0.45	294	G	0.06
115	G	0.20	295	C	0.20
116	G	0.51	296	U	0.17
117	C	0.00	297	U	0.56
118	A	0.28	298	U	0.37
119	A	0.20	299	U	ND
120	A	0.45	300	U	0.43
121	A	0.35	301	C	0.60
122	A	0.18	302	U	0.27
123	A	0.00	303	C	0.87
124	G	0.08	304	U	0.49
125	G	0.09	305	A	0.34
126	A	0.29	306	U	0.10
127	C	0.09	307	C	0.03
128	G	0.15	308	U	0.09
129	G	0.68	309	U	0.51
130	U	0.19	310	A	0.31
131	G	0.04	311	C	0.24
132	G	0.01	312	U	0.58
133	G	0.00	313	U	0.69
134	G	0.16	314	G	0.08
135	A	0.35	315	C	0.04
136	G	0.30	316	U	0.32
137	U	0.61	317	U	0.18
138	G	0.69	318	C	0.04
139	C	0.17	319	G	0.03
140	C	0.04	320	G	0.09
141	C	0.15	321	G	0.08
142	A	1.33	322	G	0.01
143	G	0.25	323	C	0.12
144	C	0.03	324	G	0.20
145	G	0.00	325	A	0.09
146	G	0.09	326	G	0.09
147	C	0.02	327	G	0.04
148	C	0.05	328	G	0.12
149	G	0.13	329	U	0.17
150	A	0.35	330	G	0.13
151	C	0.34	331	U	0.97
152	A	0.07	332	U	1.19
153	G	0.14	333	U	0.84
154	G	0.12	334	A	0.23
155	A	0.09	335	G	0.23
156	G	0.28	336	C	0.05
157	U	0.52	337	C	0.02

158	A	0.52	338	C	0.07
159	A	0.41	339	U	0.42
160	U	0.70	340	U	0.14
161	U	0.34	341	G	0.13
162	C	0.07	342	G	0.36
163	C	0.03	343	A	0.04
164	C	0.11	344	A	0.00
165	G	0.50	345	C	0.00
166	C	0.22	346	C	0.00
167	C	0.15	347	G	0.00
168	G	0.33	348	C	0.00
169	A	0.50	349	A	0.08
170	A	0.62	350	G	0.02
171	A	0.42	351	U	0.08
172	C	0.70	352	U	0.01
173	A	0.21	353	G	0.12
174	G	0.15	354	G	0.06
175	G	0.21	355	U	0.06
176	G	0.06	356	U	0.05
177	U	0.60	357	C	0.05
178	U	2.04	358	C	ND
179	U	1.28	359	U	ND
180	U	0.45			

SHAPE was performed in collaboration with the EteRNA Project on nine fragments (chunks) spanning the PSTVd genome (Supplementary Figure S2) (Lee *et al*., 2014). Reactivities reported are averaged values from five replicate experiments. G/U nucleotides highlighted in yellow were judged to be base paired by this analysis. Reactivities for nucleotides highlighted in gray were not determined (ND), including nucleotides involved in a G/U pair in the canonical PSTVd structure (61:299).

Lee, J., Kladwang, W., Lee, M., Cantu, D., Azizyan, M., Kim, H., Limpaecher, A., Gaikwad, S., Yoon, S., Treuille, T., Das, R., and EteRNA Participants (2014) RNA design rules from a massive open laboratory. *Proc. Natl. Acad. Sci. USA*, **111**, 2122-2127.

Supplementary Table S3. Summary of infection rates and progeny sequences for G/U mutants

Mutants	Local Infection rate	Systemic Infection Rate	Original Mutations	Progeny Sequences
UG7:353UU	0/10	1/10	G353U	WT
UG7:353CG	0/10	0/10	U7C	NA
UG7:353GU	0/10	0/10	U7G/G353U	NA
UG7:353UA	0/10	1/10	G353A	**A271G**
UG15:347UU	10/10	10/10	G347U	G347U
UG15:347CG	0/10	1/10	U15C	**G347U**
UG15:347GU	0/10	1/10	U15G/G347U	G347U
UG15:347UA	10/10	4/10	G347A	**C228U**/G347A/**350GΔ**
UG27:335UU	10/10	0/10	G335U	NA
UG27:335CG	0/10	0/10	U27C	NA
UG27:355GU	0/10	0/10	U27G/G335U	NA
UG27:355UA	0/10	0/10	G335A	NA
UG35:326UU	0/10	0/10	G326U	NA
UG35:326CG	0/10	1/10	U35C	WT
UG35:327GU	0/10	0/10	U35G/G326U	NA
UG35:326UA	10/10	9/10	G326A	G326A
UG40:321UU	3/10	0/10	G321U	NA
UG40:321CG	8/10	9/10	U40C	U40C
UG40:321GU	10/10	0/10	U40G/G21U	NA
UG40:321UA	10/10	2/10	G321A	**99AΔ/G16A**
GU44:317UU	0/10	1/10	G44U	WT
GU44:317GC	10/10	2/10	U317C	WT
GU44:317UG	10/10	8/10	G44U/U317G	G44U/**U317A**
GU44:317AU	8/10	10/10	G44A	G44A/**99AΔ/117A118**
GU49:312UU	10/10	7/10	G49U	G49U
GU49:312GC	10/10	2/10	U312C	WT
GU49:312UG	10/10	3/10	G49U/U312G	WT
GU49:312AU	10/10	4/10	G49A	G49A
GU61:299UU	8/10	0/10	G61U	NA
GU61:299GC	0/10	1/10	U299C	WT
GU61:299UG	0/10	0/10	G61U/U299G	NA
GU61:299AU	10/10	1/10	G61A	WT
GU64:296UU	10/10	2/10	G64U	G64U
GU64:296GC	0/10	0/10	U296C	NA
GU64:296UG	10/10	0/10	G64U/U296G	NA
GU64:296AU	0/10	0/10	G64A	NA
GU76:283UU	7/10	0/10	G76U	NA

GU76:283GC	7/10	1/10	U283C	U283C
GU76:283UG	4/10	0/10	G76U/U283G	NA
GU76:283AU	10/10	0/10	G76A	NA
UG104:254UU	10/10	10/10	G254U	G254U
UG104:254CG	0/10	1/10	U104C	**C348U**
UG104:254GU	4/10	2/10	U104G/G254U	**G44A**/**A173G**
UG104:254UA	10/10	10/10	G254A	G254A
GU106:252UU	10/10	2/10	G106U	G106U
GU106:252GC	10/10	8/10	U252C	**G199A**/U252C/**G353A**
GU106:252UG	10/10	0/10	G106U/U252G	NA
GU106:252AU	10/10	10/10	G106A	G106A
UG114:246UU	8/10	7/10	G246U	G246U
UG114:246CG	10/10	7/10	U114C	U114C
UG114:246GU	10/10	2/10	U114G/G246U	**114UΔ**/G246U
UG114:246UA	10/10	0/10	G246A	NA
GU115:245UU	10/10	9/10	G115U	G115U
GU115:245GC	10/10	6/10	U245C	U245C
GU115:245UG	8/10	0/10	G115U/U245G	NA
GU115:245AU	9/10	9/10	G115A	G115A
UG130:232UU	10/10	5/10	G232U	G232U
UG130:232CG	10/10	10/10	U130C	U130C
UG130:232GU	9/10	7/10	U130G/G232U	U130G/G232U
UG130:232UA	10/10	9/10	G232A	G232A
GU132:230UU	10/10	8/10	G132U	**A222U**
GU132:230GC	10/10	10/10	U230C	**C235A**
GU132:230UG	10/10	6/10	G132U/U230G	G132U/U230G
GU132:230AU	7/10	9/10	G132A	G132A
GU156:205UU	8/10	0/10	G156U	NA
GU156:205GC	0/10	0/10	U205C	NA
GU156:205UG	0/10	0/10	G156U/U205G	NA
GU156:205AU	10/10	0/10	G156A	NA

The 68 G/U mutants are listed, with local and systemic infection rates. Progeny obtained from pooled systemically infected leaves were sequenced. Mutants shaded in yellow retained introduced mutations and did not acquire new mutations. Sequences in bold indicate new acquired mutations. WT: reverted to wild type. NA: not applicable.

Supplementary Table S4. Conservation of G/U pairs

G/U positions on PSTVd genome	Substitutions identified in NCBI variants (n=363)	
Case 0		
15:347 UG	15U (1G,1C)	347G (none)
27:335 UG	27U (none)	335G (12U,1C)
40:321 UG	40U (none)	321G (none)
61:299 GU	61G (2A)	299U (none)
64:296 GU	64G (39U,2A)	296U (none)
104:254 UG	104U (none)	254G (7A)
106:252 GU	106G (none)	252U (none)
130:232 UG	130U (21C,1A,1G)	232G (none)
132:230 GU	132G (none)	230U (none)
Case 1		
7:353 UG	7U (none)	353G (none)
35:326 UG	35U (1C)	326G (none)
44:317 GU	44G (12U,2A)	317U (31C,4A)
115:245 GU	115G (none)	245U (1G,4A)
Case 2		
49:312 GU	49G (12A)	312U (29G,6A)
76:283 GU	76G (none)	283U (none)
156:205 GU	156G (none)	205U (none)
114:246 UG	114U (none)	246G (none)

A total of 363 PSTVd variants obtained from the National Center for Biotechnology Information (NCBI) (Ribovaria; https://www.ncbi.nlm.nih.gov/genomes/GenomesGroup.cgi?taxid=2559587) were evaluated for variability at the 17 G/U positions, grouped according to context (Black, Case 0; Red, Case 1; Blue, Case 2. Substitutions at each individual base are listed in right hand columns, with type and numbers of substitutions in parentheses. Sequences at most sites are highly conserved, and six G/U pairs were invariant: 40:321, 132:230 (Case 1), 7:353 (Case 1), 76:283, 156:205, 114:246 (Case 2).
